# Fragile Site Instability in *Saccharomyces cerevisiae* Causes Loss of Heterozygosity by Mitotic Crossovers and Break-Induced Replication

**DOI:** 10.1371/journal.pgen.1003817

**Published:** 2013-09-19

**Authors:** Danielle M. Rosen, Ellen M. Younkin, Shaylynn D. Miller, Anne M. Casper

**Affiliations:** Department of Biology, Eastern Michigan University, Ypsilanti, Michigan, United States of America; Columbia University, United States of America

## Abstract

Loss of heterozygosity (LOH) at tumor suppressor loci is a major contributor to cancer initiation and progression. Both deletions and mitotic recombination can lead to LOH. Certain chromosomal loci known as common fragile sites are susceptible to DNA lesions under replication stress, and replication stress is prevalent in early stage tumor cells. There is extensive evidence for deletions stimulated by common fragile sites in tumors, but the role of fragile sites in stimulating mitotic recombination that causes LOH is unknown. Here, we have used the yeast model system to study the relationship between fragile site instability and mitotic recombination that results in LOH. A naturally occurring fragile site, FS2, exists on the right arm of yeast chromosome III, and we have analyzed LOH on this chromosome. We report that the frequency of spontaneous mitotic BIR events resulting in LOH on the right arm of yeast chromosome III is higher than expected, and that replication stress by low levels of polymerase alpha increases mitotic recombination 12-fold. Using single-nucleotide polymorphisms between the two chromosome III homologs, we mapped the locations of recombination events and determined that FS2 is a strong hotspot for both mitotic reciprocal crossovers and break-induced replication events under conditions of replication stress.

## Author Summary

Loss of heterozygosity (LOH) at tumor-suppressor genes contributes to cancer, and deletions resulting in LOH are frequently observed in tumor cells at certain chromosomal regions known as common fragile sites. LOH can also result from repair of DNA damage by mitotic recombination, if the homologous chromosome rather than the sister chromatid is used as a repair template. The extent to which fragile site instability causes LOH by mitotic recombination with the homologous chromosome is unknown. We evaluated mitotic recombination on the yeast *Saccharomyces cerevisiae* chromosome III, which contains a naturally-occurring fragile site known as FS2. We report that yeast chromosome III has a high frequency of spontaneous mitotic recombination that involves the homologous chromosome. Under conditions that stimulate instability at the fragile site, LOH resulting from mitotic recombination on yeast chromosome III is increased 12-fold, and FS2 is a hotspot for initiating these events. These results suggest that instability at human common fragile sites may drive mitotic recombination repair pathways that cause LOH and promote tumorogenesis.

## Introduction

Cancer cells contain a variety of genomic changes that result in altered gene expression affecting cell growth. Amplification or over-expression of oncogenes and loss of heterozygosity (LOH) at tumor-suppressor genes are both significant contributors to tumorogenesis. Human common fragile sites have been extensively investigated for their contribution to genomic changes that cause tumor initiation and progression. Common fragile sites are large genomic regions of 250 kb–1 Mb that are unstable under conditions that partially inhibit DNA replication (reviewed in [1]). Treatment with aphidicolin, which inhibits DNA polymerases [2], [3], or hydroxyurea, which inhibits ribonucleotide reductase and results in unbalanced nucleotide pools [4], both cause replication stress that induces instability at fragile sites. Several mechanisms have been proposed to explain why breaks form in human common fragile sites, including secondary structure formation within single-stranded DNA (ssDNA) at stalled replication forks [5], [6], paucity of replication origins [7], [8], replication fork pausing between early- and late-replicating regions [9], [10], and collision between RNA and DNA polymerases [11]. Multiple mechanisms may contribute to breaks, and each mechanism may be responsible for breaks at a particular site or group of sites. The mutations at common fragile sites appear to often be early drivers of tumorogenesis rather than later “passenger” events [12]–[14]. This may be because replication stress resulting from nucleotide deficiency and oncogene-induced hyper replication occurs early in the progression of cancer [15]–[17].

Research to date has focused on the ability of common fragile sites to cause deletions at tumor-suppressor genes, initiate oncogene amplification by breakage-fusion-bridge cycles, generate non-reciprocal translocations, and promote integrations of human papilloma virus (reviewed in [18]). However, common fragile sites are also hotspots for sister chromatid exchange [19], and down-regulation of Rad51 in human cells, a key protein in homologous recombination, results in increased gaps and breaks at common fragile sites [20], which suggests the potential for fragile site lesions to also cause LOH through homologous recombination. Double-strand breaks are the canonical inducer of homologous recombination, but this repair pathway can also be stimulated by single-strand gaps and stalled replication forks, lesions that are likely to occur at fragile sites [21]–[23]. Homologous recombination in mitosis favors use of the sister chromatid as a repair template and use of non-crossover resolution pathways [24]–[27], but inter-homolog events can occur and result in LOH from crossovers, break-induced replication (BIR), and local gene conversion events [28], [29]. Mitotic recombination events that cause LOH have been understudied, and it is unknown to what extent the replication stress present early in cancer development causes LOH by mitotic recombination, and whether fragile sites contribute to these events.

In *Saccharomyces cerevisiae*, a fragile site named FS2 was identified on chromosome III [30]. FS2 is composed of two, 6 kb Ty1 elements in inverted orientation separated by ∼280 bp. Like human fragile sites, FS2 is a hotspot for double-strand breaks under conditions of DNA replication stress when DNA polymerases are partially impeded [30], [31]. In cells with normal levels of polymerase, FS2 is more stable but it is a hotspot for BIR events leading to non-reciprocal translocations between Ty1 elements, indicating that the fragile site is active even in the absence of replication stress [30], [32], [33]. In cells with low levels of DNA polymerase, it is likely that long stretches of single-stranded DNA form at the replication fork, which we hypothesize allows the inverted Ty1 elements of FS2 to self-pair into a hairpin structure, and cleavage of this hairpin results in a DSB [30], [34]. Here, we have used this yeast model to examine the role of fragile site instability in stimulating LOH during mitosis. In diploid cells, we determined the frequency of mitotic recombination events on chromosome III occurring spontaneously and under conditions of replication stress by low levels of polymerase alpha. Frequent single-nucleotide polymorphisms (SNPs) between the two chromosome III homologs were used to map the location of crossovers and BIR events. We find that chromosome III has a higher than expected level of spontaneous mitotic BIR, compared to reports for chromosomes IV, XII, and XV [35]–[37], and that replication stress elevates mitotic recombination by 12-fold. Reciprocal crossovers and BIR events occur at approximately equal frequencies under replication stress, and fragile site FS2 is a strong hotspot for causing LOH by both of these types of events. Our analysis of gene conversions associated with crossovers indicates that lesions at FS2 during replication, and not during G1, are the primary stimulation for these mitotic recombination events.

## Results

### Experimental system for analysis of mitotic events on yeast chromosome III

The naturally-occurring fragile site FS2 is located on *S. cerevisiae* chromosome III [30]. To evaluate mitotic recombination stimulated by this fragile site, we constructed diploids based on the detection system developed to study mitotic crossovers on yeast chromosome V [38], [39] (also see Text S1). An event that causes loss of heterozygosity at the *SUP4-o* locus in a mitotic division at the time of plating results in a red/white or red/light pink sectored colony. Therefore by their nature, each sectored colony represents an independent event.

**Figure 1:**
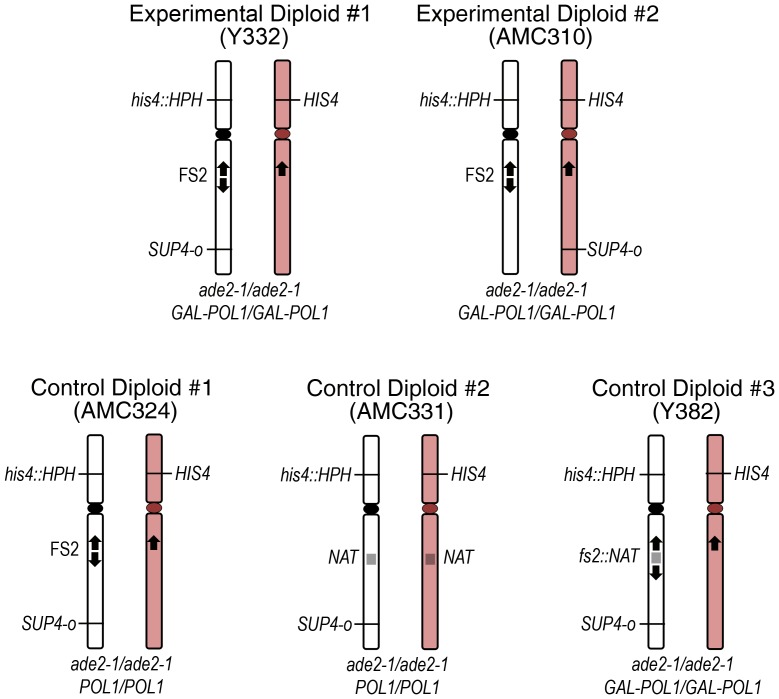
Diploids used for analysis of mitotic recombination events. Five diploid strains were used to detect events that result in loss of heterozygosity (LOH) at the *SUP4-o* locus. Only chromosome III is depicted. The white chromosome represents the MS71-derived homolog and the pink chromosome represents the YJM789-derived homolog. Experimental Diploids #1 and #2 both contain fragile site FS2, and are homozygous for the *GAL-POL1* construct that permits induction of replication stress by low levels of polymerase alpha. Experimental Diploids #1 and #2 differ in which homolog of chromosome III carries the single copy of *SUP4-o* that is present in the strain. Control Diploid #1 is isogenic to Experimental Diploid #1 except that the *POL1* gene is under its native promoter. Control Diploid #2 is isogenic to Control Diploid #1 except that the entire FS2 region has been replaced by the *NAT* drug resistance gene. Control Diploid #3 is isogenic to Experimental Diploid #1 except that fragile site FS2 has been stabilized by insertion of the *NAT* gene between the pair of inverted Ty1 elements at this site.

The relevant features of the five diploid strains we created are shown in Figure 1. These strains are homozygous for *ade2-1*, which is an ochre stop codon null mutant allele. Cells with mutant *ade2* are adenine auxotrophs and appear red due to a build-up of a red precursor in the metabolic pathway for adenine synthesis. We inserted a single copy of the tRNA ochre suppressor *SUP4-*o on the right arm of one homolog of chromosome III approximately 159 kb distal to the centromere. *SUP4-*o suppresses the ochre stop mutation; therefore the diploids are adenine prototrophs and light pink in color. The diploids are also homozygous for the *GAL-POL1* construct, except for strains AMC324 and AMC331 [30], [34]. This construct links the *GAL1/10* promoter to the *POL1* gene, so that the level of Pol1p in the cell is regulated by galactose in the growth medium, which allows us to induce replication stress and instability at FS2. Under high galactose conditions (0.05%), the level of Pol1p is approximately 300% of wild-type levels, and under low galactose conditions (0.005%), it is limited to approximately 10% of wild-type levels, thereby putting the cell under replication stress [30].

**Figure 2:**
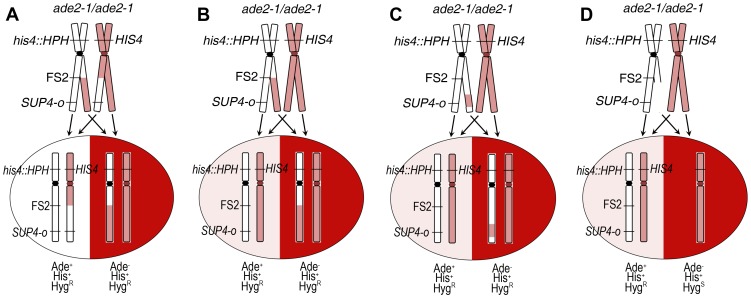
Detection of mitotic events by sectored colony formation. Mitotic events that result in loss of heterozygosity (LOH) at the *SUP4-o* locus in Experimental Diploid #1 are shown. Only chromosome III is depicted; white represents the MS71-derived homolog and pink represents the YJM789-derived homolog. The *SUP4-o* gene is approximately 159 kb from the centromere on the MS71-derived chromosome III homolog. This diploid is homozygous *ade2-1*/*ade2-1*. This mutation is suppressible by the *SUP4-o* tRNA, therefore all starting diploids are light pink in color. (A) A reciprocal crossover between the centromere of chromosome III and the *SUP4-o* locus that occurs at the time of plating results in red/white sectoring. Only half of crossover events are detected as a sectored colony, due to the pattern of sister chromatid segregation. The segregation pattern that results in sectoring is shown. (B) A break-induced replication (BIR) event that is initiated by damage centromere-proximal to *SUP4-o*, and in which the homolog that does not contain *SUP4-o* is used as a template for copying, produces a red/light pink sectored colony. (C) A local gene conversion event at the *SUP4-o* locus in which genetic information is copied from the homolog that does not contain *SUP4-o* results in red/light pink sectoring. A point mutation at the *SUP4-o* locus (not shown) will also produce a red/light pink sectored colony. (D) Damage on the homolog containing *SUP4-o* that results in chromosome loss, or deletion of the right arm of the chromosome (not shown), produces a red/light pink sectored colony.

Sectored colonies can result from several types of events that cause loss of heterozygosity at the *SUP4-o* locus in our diploids: crossover, BIR, local gene conversion, and chromosome loss. A crossover between the chromosome III centromere and *SUP4-o* is diagrammed in Figure 2A. As shown, one daughter cell is homozygous *SUP4-o/SUP4-o*, and the other daughter cell lacks this gene. Only half of crossover events are detected, due to chromosome segregation patterns. If the two recombined chromosomes segregate together in cell division, no red/white sectoring will occur. The two possible segregation patterns are equally likely in yeast [40] therefore the frequency of crossovers observed in our experiments is multiplied by two to obtain the total frequency of crossovers. Sectoring can also result from a BIR event initiated between the centromere and *SUP4-o* that proceeds centromere-distal from invasion, local gene conversion at *SUP4-o*, or loss of the chromosome containing *SUP4-o* (Figures 2B, 2C, and 2D), although in these cases the sectoring is red/light pink. BIR initiated by a lesion in the homolog that does not contain *SUP4-o* results in white/light pink sectoring. This color difference is difficult to consistently detect, and therefore white/light pink sectors were not examined. A BIR event initiated on the right arm that proceeds centromere-proximal would not be detected; however, this type of event is unlikely because BIR is impeded by the centromere [41]. Loss of Hyg^R^ in the red side of a sectored colony suggests chromosome loss (Figure 2), although BIR or crossover on the left arm of chromosome III can affect this phenotype. A point mutation in *SUP4-o* also results in red/light pink sectoring (not shown).

Our diploids have ∼0.5% sequence divergence between homologous chromosomes, as a result of mating a haploid derived from YJM789 with an S228c-related haploid [42]. This divergence in sequence does not cause a significant change in the rate of mitotic crossovers [39]. In our diploids, the S228c-related haploid is MS71, and it contains fragile site FS2 on chromosome III [31]. The YJM789-derived chromosome III does not contain FS2; therefore, to provide homology for recombination, we inserted one Crick-orientation Ty1 element in the corresponding location on this chromosome. We used single nucleotide polymorphisms (SNPs) between homologs that change a restriction enzyme site to map and analyze recombination events.

Because BIR events and chromosome loss are readily detectable in our system as red/light pink sectors only when the initiating lesion occurs on the homolog containing *SUP4-o*, we created two different experimental diploid strains (Figure 1). In Experimental Diploid #1, both *SUP4-o* and FS2 are on the MS71-derived homolog of chromosome III, which allows us to evaluate the frequency of BIR and chromosome loss from initiating lesions on this homolog. To evaluate BIR and chromosome loss that result from initiating lesions on the YJM789-derived chromosome III, which does not contain FS2, we created Experimental Diploid #2 (strain AMC 310) by moving *SUP4-o* to the YJM789-derived homolog.

### Frequency of spontaneous mitotic events resulting in LOH on chromosome III

Spontaneous mitotic events on chromosome III were initially evaluated in Experimental Diploid #1 (Y332) grown in medium with high galactose. This diploid is homozygous for the *GAL-POL1* construct and the single copy of *SUP4-o* is located on the same homolog of chromosome III as fragile site FS2 (Figure 1). We identified 31 sectored colonies among 30,543 total colonies. The event responsible for each sectored colony was determined through a combination of phenotype analysis and SNP genotyping, and frequencies for all event classes are reported in Table 1.

**Table 1:** Instability at fragile site FS2 stimulates LOH by mitotic recombination and chromosome loss.

				Number of sectored colonies			Frequency of mitotic events causing LOH (×10^−5^)			
Strain^a^	Description	Treatment	Total colonies	Crossover	BIR	Chr loss	All events	Crossover^b^	BIR^c^	Chr loss^d^
Experimental Diploid #1 (Y332)	FS2, *SUP4-o* on same chr III homolog	High gal	n = 30,543	4	14	13	115 (80–160) [1]^e^	26 (10–60) [1]	46 (30–80) [1]	43 (20–70) [1]
Experimental Diploid #1 (Y332)	FS2, *SUP4-o* on same chr III homolog	No gal	n = 22,640	29	66	45	747 (640–870) [7]	256 (200–340) [10]	292 (230–370) [6]	199 (150–270) [5]
Experimental Diploid #2 (AMC310)	FS2, *SUP4-o* on opposite chr III homologs	No gal	n = 14,876	15	28	4	417 (330–540) [4]	202 (140–290) [8]	188 (130–280) [4]	27 (10–80) [0.6]
Control Diploid #1 (AMC324)	Native *POL1*; isogenic with Y332	High gal	n = 31,810	5	8	2	62 (40–100) [0.5]	31 (10–60) [1]	25 (10–60) [0.5]	6 (0–30) [0.1]
Control Diploid #2 (AMC331)	Native *POL1*; FS2 deleted; isogenic with Y332	High gal	n = 31,182	1	9	6	54 (30–80) [0.5]	6 (0–30) [0.2]	29 (10–60) [0.6]	19 (10–50) [0.4]
Control Diploid #3 (Y382)	FS2 stabilized; isogenic with Y332	No gal	n = 21,666	3	4	0	47 (30–90) [0.4]	28 (10–70) [1]	19 (10–50) [0.4]	0 (0–20)

a All diploids result from mating a MS71-derived haploid with a YJM789-derived haploid. Each haploid is isogenic with its parent strain, except for changes introduced by transformation (described in Table S1). MS71 is a *LEU2* derivative of AMY125 (*MATα ade5-1 leu2-3 trp1-289 ura3-52 his7-2*) [64]. YJM789 is a derivative of a clinical yeast isolate (*MAT*a *ura3 gal2 ho::hisG*) [42]. All diploids except AMC324 and AMC331 are homozygous *GAL-POL1*.

b The frequency of reciprocal crossovers was calculated as 2*(number of crossover events/total colonies).

c Only BIR events that result from a break on the chromosome containing the *SUP4-o* allele can be detected as a red/light pink sectored colony. Therefore, in Y332, Y382, AMC324, and AMC331, we report BIR resulting from breaks on the MS71-derived chromosome III. In AMC310, we report BIR resulting from breaks on the YJM789-derived chromosome III.

d Only loss of the chromosome containing the *SUP4-o* allele can be detected as a red/light pink sectored colony. Therefore, in Y332, Y382, AMC324, and AMC331, we report loss of the MS71-derived chromosome III. In AMC310, we report loss of the YJM789-derived chromosome III.

e The number in parenthesis is the 95% confidence interval. The number in brackets is the fold change from Y332 in high galactose. No local gene conversion at the *SUP4-o* locus was observed in any strain; therefore this category is not included in the table.

In Experimental Diploid #1 on high galactose, the total frequency of spontaneous mitotic events resulting in LOH on the right arm of chromosome III is 115×10^−5^. We observed three categories of events: reciprocal crossovers, BIR, and chromosome loss. The spontaneous frequency of crossovers is 26×10^−5^. Since the interval between *CEN3* and *SUP4-o* is 159 kb, this is 1.65×10^−6^ crossovers per kb. The frequency of spontaneous BIR events initiated between *CEN3* and *SUP4-o* is 46×10^−5^, or 2.89×10^−6^ BIR events per kb. Because BIR is unidirectional in its transfer of genetic information, only BIR initiated by a lesion in the homolog containing *SUP4-o* results in red/light pink sectoring (Figure 2). Initiating lesions on the other homolog that are repaired by BIR result in white/light pink sectoring that is not easily detected. Therefore, BIR in Experimental Diploid #1 reported in Table 1 reflects only events initiated by a break on the MS71-derived homolog of chromosome III, which contains both *SUP4-o* and fragile site FS2. Similarly, loss of the *SUP4-o* containing homolog of chromosome III is detectable by red/light pink sectoring. The frequency of spontaneous loss of the MS71-derived chromosome III is 43×10^−5^ in Experimental Diploid #1.

Cells with *GAL-POL1* grown on high galactose contain an excess of Pol1p and have a modest increase in instability at fragile site FS2 relative to strains with *POL1* under its native promoter [30]. To evaluate the effect of excess Pol1p on mitotic recombination, we created Control Diploid #1, which is isogenic to Experimental Diploid #1 but homozygous for *POL1* under its native promoter. After growth in medium with high galactose, the total frequency of spontaneous mitotic events resulting in LOH is reduced by half in this control diploid compared to Experimental Diploid #1 on high galactose (*p* = 0.0187) (Table 1). However, the relative proportions of each type of mitotic event (crossover, BIR, and chromosome loss) are not significantly different between these two diploids (*p* = 0.089).

FS2 is a hotspot for Ty1-mediated translocations under normal polymerase conditions [32], [33]. To evaluate the effect of FS2 instability on mitotic recombination in cells with normal levels of *POL1*, we modified Control Diploid #1 by replacing the entire FS2 region on the MS71-derived homolog, including both Ty1 elements and the nucleotides between them, with the *NAT* gene [43]. The same region on the YJM789-derived homolog was also replaced with the *NAT* gene. This diploid is referred to as Control Diploid #2. We found that there is no difference in the total frequency of spontaneous mitotic LOH events on the right arm of chromosome III between Control Diploids #1 and #2 after growth in medium with high galactose (*p* = 1.0) (Table 1).

### Increased chromosome III mitotic crossovers, BIR, and chromosome Ioss in cells under replication stress

Partial inhibition of replication by lowering the level of DNA polymerase alpha causes breaks on yeast chromosome III at fragile site FS2. In haploid cells these breaks are frequently repaired by BIR or result in loss of chromosome III and can be detected by increased illegitimate mating [30], [34]. In diploid cells, low polymerase alpha increases mitotic reciprocal crossovers within the yeast rDNA array by 7-fold [44]. Here, we have further evaluated the role of replication stress in stimulating events that cause LOH in diploid cells, and the role of fragile site instability in initiating these events. To study stress-induced mitotic events on yeast chromosome III, we grew Experimental Diploid #1 in medium with no galactose for six hours to lower the level of polymerase alpha, followed by plating on high galactose. We identified 140 sectored colonies among 22,640 total colonies. Replication stress in this diploid increases the total frequency of mitotic LOH events by 6.5-fold relative to high galactose conditions (*p*<0.001) (Table 1). However, the relative proportions of the categories of crossover, BIR, and chromosome loss in this diploid are the same in both high galactose and no galactose (*p* = 0.463).

As explained above, BIR events and chromosome loss are readily detectable in our system only when the initiating lesion occurs on the homolog containing *SUP4-o*. In Experimental Diploid #1, both FS2 and *SUP4-o* are on the MS71-derived homolog. We used Experimental Diploid #2, which has *SUP4-o* on the non-FS2 containing YJM789-derived homolog, to evaluate the frequency of BIR and chromosome loss from initiating lesions on this homolog of chromosome III. We grew this diploid in medium lacking galactose for six hours to induce replication stress then plated cells on high galactose. We identified 47 sectored colonies among 14,876 total colonies. Under replication stress, the total frequency of mitotic LOH on chromosome III in Experimental Diploid #2 is half that of Experimental Diploid #1 (*p*<0.001) (Table 1). The frequency of crossovers is similar in Experimental Diploids #1 and #2 under replication stress (*p* = 0.543) (Table 1). This result is consistent with our expectation, given that crossovers are detected in our system irrelevant of which chromosome III homolog contains the initiating lesion. The frequency of replication stress-induced BIR is one-third lower in Experimental Diploid #2 than in Experimental Diploid #1 (*p* = 0.0629). This difference results from the absence of FS2 in the *SUP4-o* marker homolog. In Experimental Diploid #2, only BIR events that are initiated by lesions on the YJM789-derived chromosome can be detected as red/light pink sectors. Events caused by a lesion at FS2 on the MS71-derived homolog of chromosome III will result in white/light pink sectoring, which is not easily detected and thus not scored in this diploid. The lower frequency of BIR in Experimental Diploid #2 suggests that FS2 instability drives 1/3 of the stress-induced BIR observed in Experimental Diploid #1. There is an even stronger reduction in the frequency of chromosome III loss in Experimental Diploid #2 under replication stress, such that the loss frequency is below that observed in Experimental Diploid #1 with high levels of polymerase. Therefore, the primary cause of chromosome III loss in Experimental Diploid #1 is FS2 instability.

To further evaluate the role of FS2 instability in driving mitotic LOH under replication stress, we created Control Diploid #3 (Y382), in which we stabilized fragile site FS2. Normally, the inverted Ty1 elements at FS2 are separated by ∼280 bp. We inserted the *NAT* gene [43] between these two Ty1 elements, separating them by ∼1.8 kb. The increased distance effectively stabilizes the fragile site [30]. We grew Control Diploid #3 in medium with no galactose for six hours to induce replication stress conditions, and then plated cells on high galactose medium. We identified 7 sectored colonies among 21,666 total colonies, and analyzed these sectored colonies as before. Under replication stress, the total frequency of mitotic LOH on chromosome III in Control Diploid #3 is less than half that of Experimental Diploid #1 (*p*<0.0001) (Table 1). However, the relative proportions of the categories of crossover, BIR, and chromosome loss events in these diploids is similar (*p* = 0.1106).

### Fragile site FS2 is a hotspot for replication stress-induced BIR events

**Figure 3:**
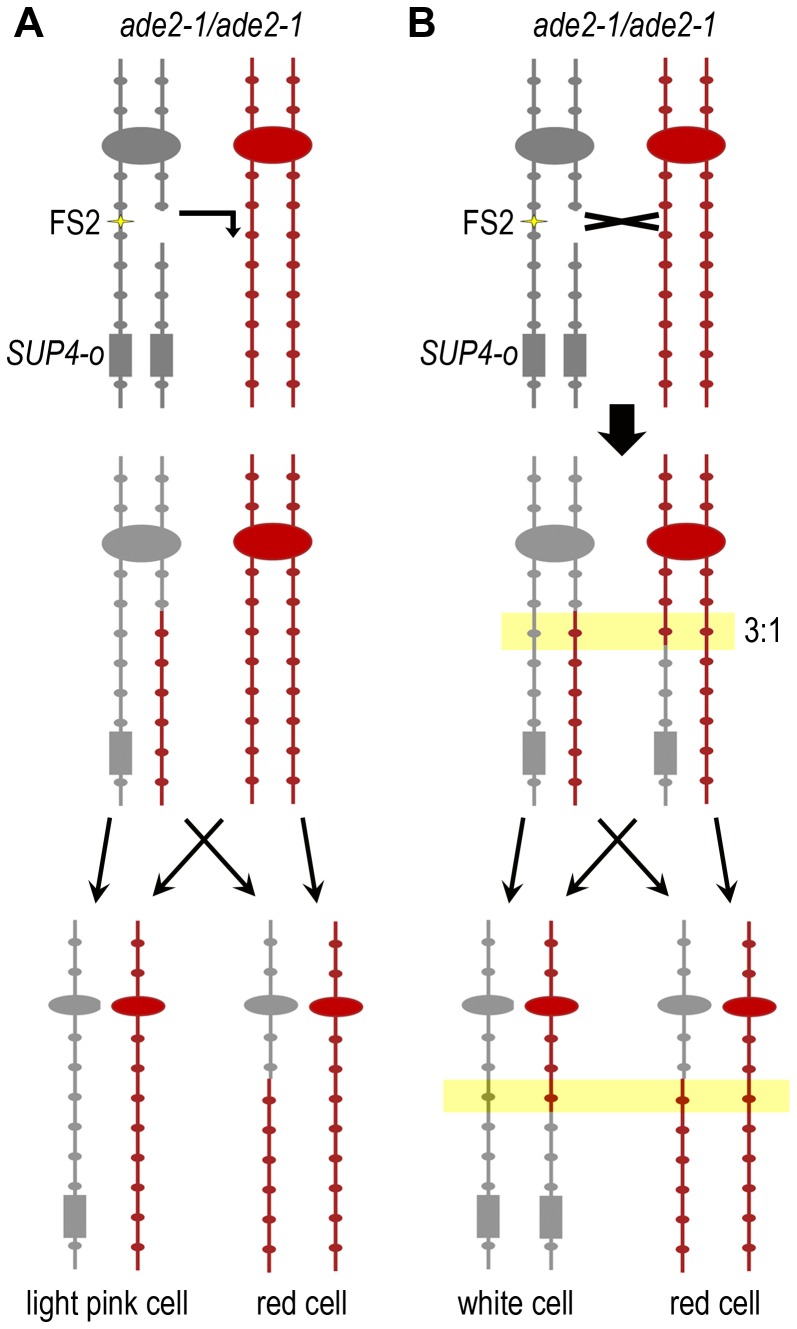
Use of SNPs to map the location of mitotic recombination events. Single nucleotide polymorphisms (SNPs) between the two homologs of chromosome III that alter restriction sites were used to evaluate the type of event responsible for sectoring and to map the location of each event. Experimental Diploid #1 is shown. The gray chromosome represents the MS71-derived homolog and the red chromosome represents the YJM789-derived homolog. Centromeres are represented by large ovals and SNP sites by small ovals. FS2 is indicated by yellow stars and *SUP4-o* is represented by a gray rectangle. This strain is homozygous for the ochre-suppressible *ade2-1* mutation. (A) A BIR event that is stimulated by a lesion at FS2 is shown. The YJM789-derived homolog is used as a template for repair. After chromosome segregation in mitosis, the light pink cell remains heterozygous at all SNPs, while the red cell is homozygous for the YJM789 form of all SNPs distal to the invasion site. (B) A reciprocal crossover that occurs to repair a lesion at FS2 in S phase or G2 is shown. The crossover location is indicated by a black X. Transfer of genetic information from the YJM789-derived homolog during repair resulting in 3∶1 gene conversion of one SNP is shown in the yellow box. After chromosome segregation in mitosis, the white cell is homozygous for the MS71 version of SNPs distal to the crossover, while the red cell is homozygous for the YJM789 form of SNPs distal to the crossover, and the SNP within the region of gene conversion is homozygous in the red cell but heterozygous in the white cell.

We used the ∼0.5% sequence divergence between the two homologs of chromosome III in our diploids to map the locations of the events causing sectoring. This divergence results in many single nucleotide polymorphisms (SNPs) between the homologs, some of which alter restriction enzyme sites. We purified a single cell from each half of the sectored colony and evaluated a set of 27 SNPs on the right arm of chromosome III by PCR and restriction enzyme digest (Table S3). On average, these SNPs are spaced 6.9 kb apart. The closest SNP centromere-distal to FS2 that changes a restriction enzyme site is at chromosome III base 175324; this is 5.4 kb from the end of the fragile site. The closest SNP centromere-proximal to FS2 is at base 167720; this is 0.8 kb from the end of the fragile site. As shown in Figure 3A, in the case of BIR, all SNPs in the light pink cell remain heterozygous while in the red cell, SNPs proximal to the event remain heterozygous and SNPs distal to the event are homozygous for the homolog lacking *SUP4-o*. In the case of BIR events initiated by a lesion in unique sequence, we assume that invasion of the broken end into the corresponding region on the homologous chromosome results in homozygosity for SNPs distal to the BIR. However, if the broken end occurs near a Ty1 element, it may invade a Ty1 or Ty2 element on a non-homologous chromosome to initiate replication. In such cases of non-allelic repair, SNPs distal to the BIR would be hemizygous.

**Figure 4:**
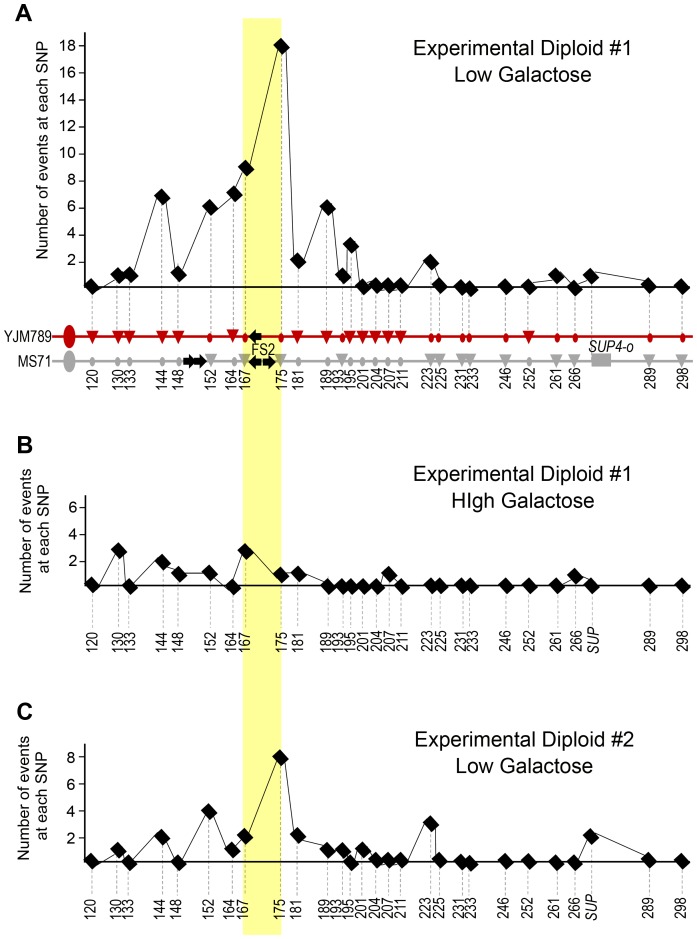
Fragile site FS2 is a hotspot for initiation of BIR events resulting in LOH. The number of events initiated at each SNP is shown, and the two SNPs flanking fragile site FS2 are highlighted with a yellow box. The SNP indicated where each event maps is the first homozygous SNP in the stretch of homozygous SNPs. The BIR initiation site can be anywhere between the last heterozygous SNP and the first homozygous SNP. (A) The two homologs of the right arm of chromosome III are shown. The gray homolog is MS71-derived and contains fragile site FS2 and the *SUP4-o* allele. The red chromosome represents the YJM789-derived homolog. Large ovals represent the centromere. Black arrows on the chromosome diagrams indicate Ty1 elements. SNP markers used to map events are shown by circles and triangles on the chromosome diagrams. Triangles indicate a restriction site exists, circles indicate lack of the site. Numbers are the approximate chromosome coordinate in kb. The 66 BIR event initiation sites collected in Experimental Diploid #1 under low galactose conditions that cause replication stress are shown in the graph above the chromosome diagram. All BIR events in this diploid had three copies of the YJM789-derived SNPs, implying that the initiating lesion occurred on the gray, FS2-containing chromosome. (B) The 14 BIR event initiation sites collected in Experimental Diploid #1 under high galactose conditions that do not cause replication stress. (C) The 28 BIR event initiation sites collected in Experimental Diploid # 2 under low galactose conditions that cause replication stress. In this diploid, *SUP4-o* is located on the red, YJM789-derived chromosome. All BIR events in this diploid had three copies of the MS71-derived SNPs, implying that the initiating lesion occurred on the red, non-FS2-containing chromosome.

In Experimental Diploid #1 we detect only BIR initiated by lesions on the MS71-derived homolog of chromosome III, which contains both *SUP4-o* and FS2. As anticipated, only YJM789-derived SNPs are present in the red cell distal to each BIR event. Our SNP mapping indicates that fragile site FS2 is a hotspot for initiation of BIR in this diploid (Figure 4A). Of 66 total BIR events under replication stress, 18 were initiated between the SNPs flanking FS2. BIR can be initiated centromere-proximal to a break location due to exonuclease processing at the break that usually exposes 3–6 kb of ssDNA [45]; therefore, the nine BIR events initiated between the pair of SNPs immediately centromere-proximal to FS2 likely also result from lesions at FS2, for a total of 27/66 events (41%) stimulated by FS2. We evaluated the significance of this distribution by dividing the *CEN3* – *SUP4-o* interval into four equal-sized bins of 39.7 kb, then counting the number of BIR events initiated within each bin. There are 16 events in bin #1 (*CEN3* to SNP152), 43 in bin #2 (SNPs 164 to193), 5 in bin #3 (SNPs 195 to 233), and 2 in bin #4 (SNPs 246 to *SUP4-o*). This distribution is significantly different from random (*p*<0.0001 by chi-square goodness-of-fit). To determine whether BIR events were allelic or non-allelic, we determined the sizes of chromosome III in a subset of 35 BIR events from Experimental Diploid #1 under replication stress. Allelic events will produce chromosome III repair products of normal size, and non-allelic events will produce a chromosome that may be smaller or larger than the normal chromosome III size. Intact yeast chromosomes from each event were separated by pulsed-field gel electrophoresis, and chromosome size was evaluated by Southern blotting with a *CHA1* probe to the right arm of chromosome III. Of events tested that were initiated between the SNPs flanking FS2 or within 6 kb proximal of FS2, 7/18 (39%) had non-allelic BIR products (Figure S1). All of the BIR events tested that were initiated more than 6 kb proximal of a Ty1 element were allelic.

In high galactose conditions that permit high levels of *POL1* transcription, the number of BIR events initiated in Experimental Diploid #1 at or within 6 kb proximal to FS2 is reduced to 4/14 (29%) (Figure 4B). This fact that this reduction is relatively modest is likely attributable to excessive polymerase alpha causing FS2 instability in this diploid, because Control Diploid #1, which has *POL1* under its native promoter, and Control Diploid #3, which has a stabilized version of FS2, do not have any BIR events initiated at or within 6 kb proximal of FS2. We note that the pair of tandem-oriented Ty1 elements centromere-proximal to FS2 on the MS71-derived homolog is not a BIR hotspot in Experimental Diploid #1 (Figure 4A), although this was a frequent site of recombination in the illegitimate mating assays previously used to study fragile sites on yeast chromosome III [30], [34]. This difference will be further discussed below.

In Experimental Diploid #2, 10/28 BIR events (36%) were initiated at or within 6 kb proximal of the location allelic to FS2 (Figure 4C). In this diploid, only events initiated by a lesion on the YJM789-derived homolog are detected. As before, we evaluated the significance of this distribution by dividing the *CEN3* – *SUP4-o* interval into four equal-sized bins of 39.7 kb, then counting the number of BIR events initiated within each bin. There are 7 events in bin #1, 15 in bin #2, 4 in bin #3, and 2 in bin #4. This distribution is significantly different from random (*p* = 0.0029 by chi-square goodness-of-fit). Therefore, despite the fact that FS2 is not present on the YJM789-derived homolog, the site allelic to this fragile site is a hotspot for initiation of BIR events. The YJM789 homolog of chromosome III contains a pair of inverted delta elements (the ∼300 bp long terminal repeat portion of Ty1 elements) at the location allelic to FS2. The spacing between these inverted deltas is the same as between the Ty1 elements of FS2. As explained above, we modified the YJM789 homolog to expand the Crick-orientation delta element to a full Ty1, to provide homology for recombination without creating a fragile site. However, the inverted delta elements also have the potential for intra-strand base pairing to form a hairpin under conditions of replication stress. Since the overall frequency of stress-induced BIR is lower in Experimental Diploid #2 than in Experimental Diploid #1, the frequency of BIR stimulated by the “full” version of FS2 is higher than that stimulated by the “delta only” version of FS2 (frequencies of 123×10^−5^ and 67×10^−5^ FS2-stimulated BIR, respectively). There were three BIR events in Experimental Diploid #2 that had adjacent gene conversion tracts; two with a 4∶0 tract (SC100 and SC104) and one with a 3∶1 tract (SC121) (Figure S2). Gene conversion associated with BIR has previously been reported, and appeared to result from repair of two double-strand breaks in the same location on both sister chromatids, [35], [36]. The 3∶1 tract observed here does not fit that mechanism, and may instead represent repair of heteroduplex mis-matches in the region of invasion for BIR initiation. The 4∶0 tracts are unusual and may represent an internal deletion prior to BIR initiation.

### Replication stress-induced crossover events are stimulated by S-phase damage at fragile site FS2

Figure 3B shows an example of the SNP pattern in a sectored colony from a reciprocal crossover on the right arm of chromosome III. For crossovers un-associated with gene conversion, SNPs proximal to the crossover remain heterozygous, and distal to the crossover, are homozygous for the homolog lacking *SUP4-o* in the red cell, and homozygous for the homolog containing *SUP4-o* in the white cell. Gene conversion that is associated with a crossover can be of two types, either a typical 3∶1 segregation in which SNPs are heterozygous in one cell and homozygous in the other (as shown in Figure 3B), or a 4∶0 pattern in which SNPs are homozygous for the same version in both the red and white cells [39]. The 3∶1 conversions appear to result from repair of damage that occurs during S-phase and 4∶0 conversions result from DNA double-strand breaks that occur during G1 that are replicated, followed by repair of both broken sister chromatids in G2 using the unbroken homolog as a template [46].

**Figure 5:**
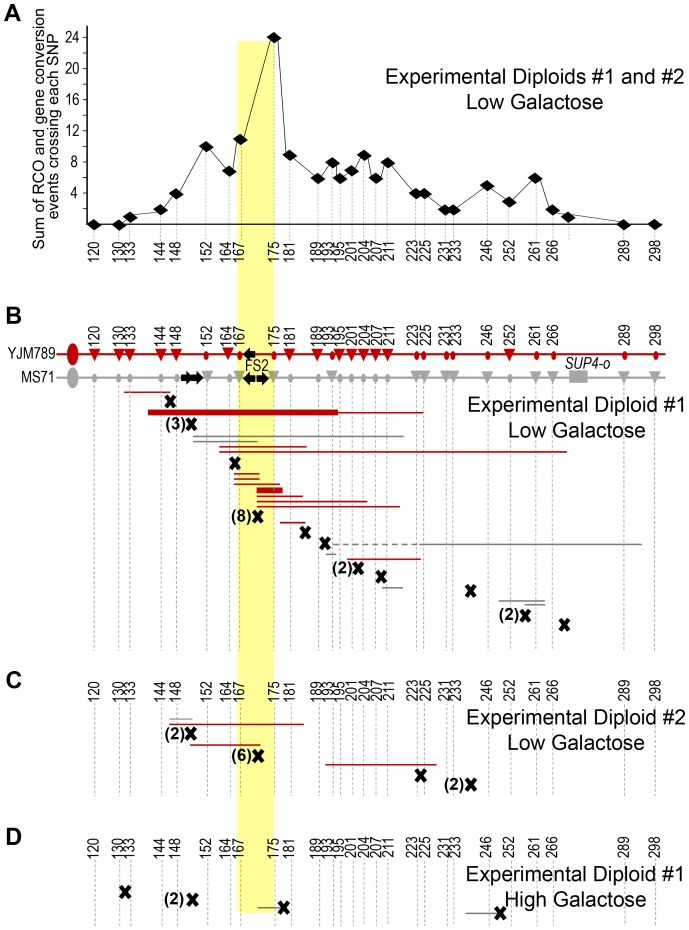
Lesions at fragile site FS2 during S-phase initiate mitotic crossovers resulting in LOH. (A) Number of events at each SNP in Experimental Diploids #1 and #2 under replication stress. We summed the number of conversion tracts (both 3∶1 and 4∶0 events) crossing each SNP in both experimental diploids, and crossovers un-associated with a gene conversion were added to the sum of the closest centromere-distal SNP. Numbers are the approximate chromosome coordinate in kb for each SNP. (B) Locations of 41 crossovers and associated gene conversions collected in Experimental Diploid #1 under replication stress. Chromosome diagrams for Experimental Diploid #1 are the same as in Figure 4. Crossover events are shown below the chromosomes. Black X's indicate crossovers that did not have gene conversion associated at the SNPs tested. A number in parenthesis indicates how many crossover events were at the site, if more than one. Thin horizontal lines indicate 3∶1 conversion tracts and thick lines indicate 4∶0 tracts. Dotted lines indicate a non-adjacent conversion tract. Line color shows which chromosome was copied in gene conversion. These crossover events were collected in two ways; 29 crossover events were collected among the colonies in Table 1, and 12 crossover events were collected among another set of 14,792 colonies. (C) Locations of the 15 crossovers and associated gene conversions collected in Experimental Diploid #2 under replication stress. In this diploid, SUP4-o is located on the red, YJM789-derived homolog of chromosome III. (D) Locations of 5 crossovers and associated gene conversions in Experimental Diploid #1 in high galactose, which permits abundant production of polymerase alpha. These crossover events were collected in two ways; four crossover events were collected among the 30,543 colonies in Table 1, and one crossover event was collected among another set of 4,792 colonies.

As shown in Figure 5A, our SNP mapping results indicate that fragile site FS2 is a hotspot for crossover events under replication stress caused by low Pol1p. We identified 41 crossover events in Experimental Diploid #1 under stress (Figure 5B). These crossover events were collected in two ways; 29 crossover events were collected among the 22,640 colonies in Table 1 that were fully analyzed for crossover, BIR, and chromosome loss events, and 12 crossover events were collected among another set of 14,792 colonies that was not fully analyzed for BIR and chromosome loss events. Of the 41 crossovers in Experimental Diploid #1, 21 have no associated gene conversion tract, 19 have a gene conversion adjacent to the crossover, and one has a conversion tract that is not contiguous with the crossover. Of the 21 crossovers without gene conversion, 8 occur between the SNPs flanking FS2. Of 19 crossovers with adjacent gene conversion tracts, 12 tracts cross a SNP flanking FS2. Therefore, 20/41 crossover events (49%) in Experimental Diploid #1 under replication stress are associated with FS2. The crossover data from Experimental Diploid #2 under replication stress is similar to Experimental Diploid #1, which is consistent with the expectation that our system detects all crossovers between *CEN3* and *SUP4-o* irrespective of which homolog contains *SUP4-o* or which homolog has the initiating lesion. In Experimental Diploid #2, 8/15 events (53%) are associated with FS2 (Figure 5B). Of 15 total crossover events, 11 are unassociated with gene conversion, and 6 of these occur between the SNPs flanking FS2. Of the 4 crossovers with adjacent gene conversion, two have tracts that cross a SNP flanking FS2; these two tracts have information transferred from the non-FS2 containing homolog indicating the initiating event was at or near the fragile site (Figure 5B).

**Figure 6:**
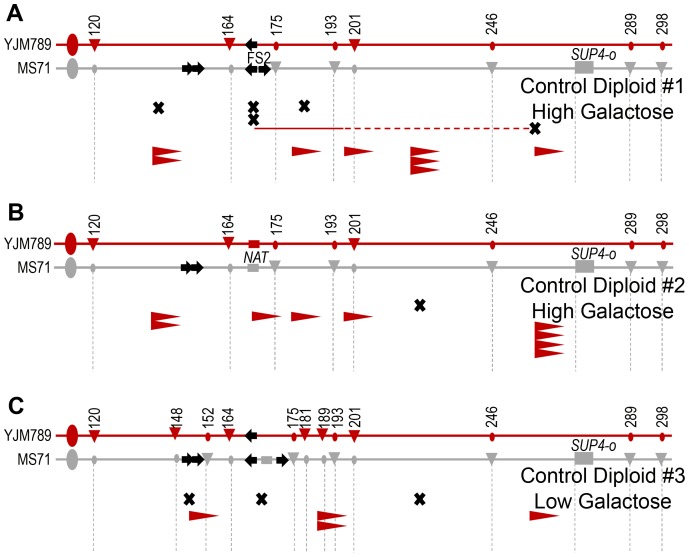
Locations of crossovers and BIR events in Control Diploids. For each diploid, the two homologs of the right arm of chromosome III are shown. The gray homolog is MS71-derived and the red homolog is YJM789-derived. Large ovals represent the centromere. Black arrows on the chromosome diagrams indicate Ty1 elements. SNP markers used to map events are shown by circles and triangles on the chromosome diagrams. Triangles indicate a restriction site exists, circles indicate lack of the site. Numbers are the approximate chromosome coordinate in kb. Crossover and BIR events are shown below the chromosomes. Black X's indicate crossovers that did not have gene conversion associated at the SNPs tested. Thin horizontal lines indicate 3∶1 conversion tracts associated with crossovers, and dotted lines indicate a non-adjacent conversion tract. Line color shows which chromosome was copied in gene conversion. BIR events are shown by arrowheads. The flat vertical edge of the arrowhead indicates the site at which the BIR was initiated; all BIR events extended to the end of the chromosome. The SNP indicated where each BIR event maps is the first homozygous SNP in the stretch of homozygous SNPs. The BIR initiation site can be anywhere between the last heterozygous SNP and the first homozygous SNP. The red color of the arrowheads indicates that the YJM789-derived homolog was the template for copying, implying that the initiating lesion occurred on the MS71-derived homolog. (A) Five crossover and eight BIR events mapped in Control Diploid #1, grown in high galactose. Both fragile site FS2 and the *SUP4-o* allele are located on the MS71-derived homolog, and this diploid is homozygous for the *POL1* gene under its native promoter. (B) One crossover and nine BIR events mapped in Control Diploid #2, grown in high galactose. This diploid is homozygous for the *POL1* gene under its native promoter, and the *NAT* gene replaces both Ty1 elements of fragile site FS2. (C) Three crossover and four BIR events mapped in Control Diploid #3 under replication stress caused by low levels of polymerase alpha. Fragile site FS2 has been inactivated in this diploid by expansion of the space between the two Ty1 elements of the fragile site.

In Experimental Diploid #1 in high galactose conditions, five crossovers were collected. These crossover events were collected in two ways; 4 crossover events were collected among the 30,543 colonies in Table 1 that were fully analyzed for crossover, BIR, and chromosome loss events, and 1 crossover event was collected among another set of 4,792 colonies that was not fully analyzed for BIR and chromosome loss events. Of these five crossovers, one is located at FS2 (Figure 5D). This crossover is associated with a gene conversion tract in which the transfer of genetic information indicates that the initiating event is on homolog with the “delta-only” FS2. In Control Diploid #1, which has *POL1* under its native promoter, two of the five crossovers are located between the nearest SNPs flanking FS2 (Figure 6). Although the number of events detected under high galactose and in Control #1 is low, it is intriguing that 20–40% of crossovers in these diploids were at FS2. We address this result in the discussion below. In Control Diploid #2, which has *POL1* under its native promoter and no FS2, the single crossover detected was not near the deleted fragile site, and in Control Diploid #3, which is *GAL-POL1* and has a stabilized version of FS2, one of the three crossovers was at FS2 (Figure 6).

Several characteristics of the crossover-associated gene conversion tracts in Experimental Diploids #1 and #2 under replication stress are of interest. First, 12 of the 14 tracts crossing a SNP at FS2 have three copies of the information from the chromosome lacking FS2. This result is consistent with damage at FS2 responsible for crossover stimulation, since in both mitotic and meiotic recombination events, the damaged chromosome typically receives genetic information from the unbroken homolog [47]–[49]. However, the two tracts that were stimulated by an initiating lesion on the YJM789-derived homolog are consistent with our BIR results above, in which a “delta-only” version of FS2 is capable of stimulating a lower level of recombination than the “full” version of FS2. Second, of 24 total tracts, only two are 4∶0 type tracts and the others are 3∶1. The 3∶1 conversions have been reported to result from repair of S-phase damage and the 4∶0 conversions result from DNA double-strand breaks in G1 that are replicated, followed by repair of both broken sister chromatids during G2 [46]. Therefore, our results indicate that the crossover-associated gene conversion tracts under replication stress are consistent with damage occurring primarily during S phase. Third, six of the gene conversion tracts associated with FS2 cross both SNPs flanking FS2, four cross only SNPs centromere-proximal, and four cross only SNPs centromere-distal. Therefore, repair of a lesion at FS2 that occurs during S-phase can result in gene conversion that extends either bi- or uni-directionally. Fourth, our mitotic gene conversion tracts are relatively long, with a median length of 14.7 kb (95% confidence interval of 7.0 kb to 34.5 kb) for the 23 tracts contiguous with a crossover. Both 4∶0 and 3∶1 tracts were included in our analysis of median tract length.

## Discussion

We have determined the frequencies of spontaneous and replication stress-induced mitotic events resulting in loss of heterozygosity (LOH) on the right arm of yeast chromosome III. Yeast fragile site FS2 is present on this chromosome, and we report that it is a hotspot for mitotic reciprocal crossovers and BIR events.

### Yeast chromosome III has a high frequency of spontaneous BIR

St Charles and Petes [28] defined the microStern (µS) as a unit to measure mitotic crossovers, with 10^−6^ crossovers/division equal to one microStern, and they estimated the entire yeast genome has a mitotic genetic map length of 620 µS. The portion of chromosome III we evaluated accounts for 1.3% of the physical yeast genome, therefore we expect a genetic map length of 8 µS. We detect a map length of 6 µS for the right arm of chromosome III in Control Diploid #2, which has normal Pol1p levels and does not contain FS2. In Control Diploid #1, which has normal Pol1p but contains FS2, we detect a map length of 310 µS, and two of the five crossover events are at FS2. These data are in accordance with reports that FS2 can be unstable under normal polymerase conditions [32], [33]. However, there is no difference in the total frequency of spontaneous mitotic LOH events between these two diploids (*p* = 1.0)(Table 1).

Previous studies of mitotic LOH in yeast have reported that BIR is less frequent than crossovers. On yeast chromosomes IV and XII, spontaneous BIR is three to four-fold less frequent than crossovers [35], [37], and on chromosome XV, repair by BIR of a mitotic double-strand break from an I-*Sce*I cut site is extremely rare compared to repair that results in crossover or non-crossover outcomes [36]. The exception to this pattern is in old yeast mother cells, in which nearly 90% of spontaneous mitotic LOH results from BIR [37]. We observed that BIR is nearly 5-fold more frequent than crossovers in Control Diploid #2, and that BIR is only 20% less frequent than crossovers in Control Diploid #1. None of the BIR events in Control Diploid #1 were initiated at or near FS2, which indicates that a mechanism other than fragile site instability drives spontaneous BIR on yeast chromosome III in this strain. The BIR pathway is primarily used to repair one-ended double-strand breaks, such as those that exist at collapsed replication forks [50]. Therefore, our results may suggest a higher frequency of spontaneous replication fork stalling and collapse on the right arm of chromosome III than on other chromosomes similarly examined to date.

### Replication stress by low levels of polymerase alpha increases mitotic recombination on yeast chromosome III

Here, we report that the total frequency of mitotic LOH is elevated 12-fold in Experimental Diploid #1 with low levels of polymerase, relative to Control Diploid #1 with wild-type levels of polymerase (Table 1). In our analysis, replication stress induces reciprocal crossovers, BIR, and chromosome loss with approximately equal frequency. In haploids with low levels of polymerase alpha, physical analysis of chromosome III indicates that a double-strand break at FS2 occurs in approximately 7% of cells [34]. If a similar percentage of diploid cells with low polymerase alpha have breaks at FS2, then our results indicate that LOH is a rare outcome in responding to these breaks. This is not unexpected, because LOH as a result of mitotic recombination requires crossover and BIR events involving the homologous chromosome. However, during mitosis the sister chromatid is favored as a repair template during S-phase [27], [51], [52], and crossover resolution of Holliday junctions is normally suppressed [53]. A related issue is the detection of gene conversion events at FS2 that are un-associated with crossover. Our system does not permit analysis of such events unless those gene conversions are large enough to also encompass *SUP4-o*. Recent studies have demonstrated that approximately 35% of conversions are crossover-associated [35], [36], [54]. Therefore, we would not expect that undetected local gene conversion events at FS2 would change the relative rarity of LOH at this site compared to the frequency of breaks.

### The inverted repeat at fragile site FS2 is a hotspot for mitotic recombination under replication stress

Here, we report that FS2 is a hotspot for driving mitotic events that result in LOH on the right arm of yeast chromosome III. Unexpectedly, a smaller inverted repeat consisting of two long terminal repeat delta elements separated by the same ∼280 bp distance as between the two full Ty1 elements of FS2, is similarly a hotspot for mitotic recombination under replication stress. However, the delta-only FS2 stimulates only half as many BIR events as the full FS2. There are no other inverted delta-delta pairs on the right arm of chromosome III to investigate for fragile site activity. However, inverted pairs of delta elements have been reported to fuse and generating acentric and dicentric chromosomes in yeast when replication is impeded; faulty template switching at stalled replication forks was proposed as a mechanism to generate these rearrangements [55]. Human *Alu* sequences, which are similar in length to the yeast delta element, stimulate breakage and recombination when inserted in inverted orientation on yeast chromosome II, although this is strongly influenced by the distance between the repeats [56].

**Figure 7:**
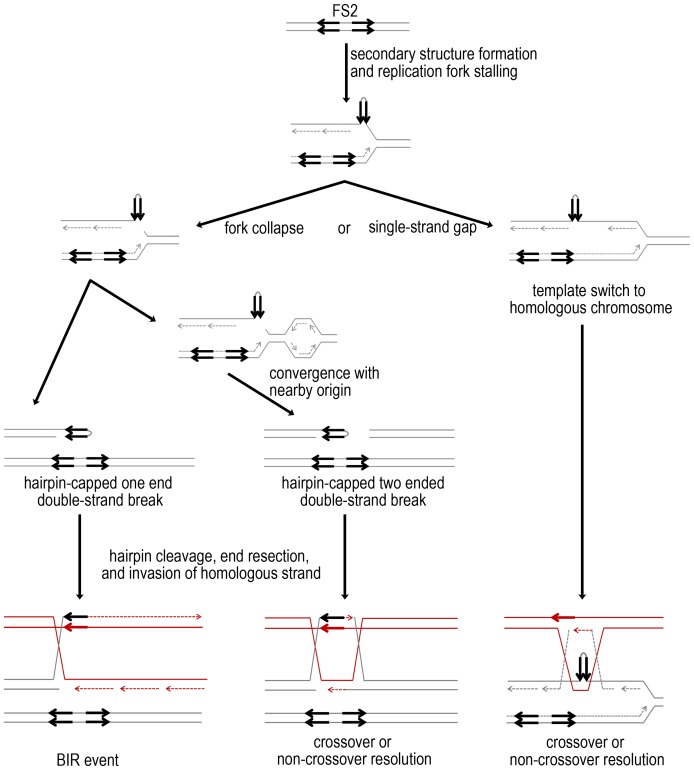
Mechanisms for fragile site stimulated mitotic crossovers and BIR events. In cells with low levels of polymerase alpha, slowed DNA replication likely results in larger regions of single-stranded DNA on the lagging strand, which allows intra-strand pairing at FS2 to form a hairpin. Here, the MS71-derived homolog of chromosome III is depicted in gray, with the two inverted Ty1 elements of FS2 as black arrows. Stalling could be followed by either cleavage at the secondary structure, resulting in fork collapse, or replication may proceed and leave a single-strand gap at the secondary structure. Fork collapse to a one-ended double-strand break is expected to primarily stimulate repair by BIR. Here, the homologous YJM789-derived chromosome III is shown in red as a repair template, resulting in LOH. If a replication from a nearby origin converges with the collapsed fork, a double-strand break will form. This lesion can be repaired by invasion of the red, YJM789-derived homolog, and capture of the second end, as shown. Crossover resolution of the double Holliday junction can result in LOH after mitotic chromosome segregation. If replication stalling results in formation of a single-strand gap rather than a break, template switching may be used to fill the gap. Template switch to the red, YJM789-derived homolog forming a double Holliday junction is shown. Crossover resolution followed by mitotic chromosome segregation can result in LOH.

It is somewhat surprising that crossovers and BIR events are stimulated approximately equally at FS2. Under replication stress, it is hypothesized that extended single-stranded DNA at the replication fork allows a hairpin to form between the pair of inverted Ty1 elements of FS2 [30], [34], and cleavage at this secondary structure would result in replication fork collapse to a one-ended double-strand break, which should primarily drive BIR (Figure 7) [50]. Crossover formation requires a double Holliday junction intermediate. The stimulation of crossovers at FS2 is primarily replication-dependent, because the gene conversion tracts adjacent to crossovers are nearly all of the 3∶1 type. Two possible ways that a double Holliday junction intermediate could form at FS2 are (1) template switching at a stalled replication fork or single-strand gap left at FS2 during replication, or (2) convergence of a collapsed fork with replication from a nearby origin, producing a canonical double-strand break (Figure 7) [22], [23]. Although physical analysis of chromosome III demonstrates that double-strand breaks do form at FS2 under replication stress [34], it is unclear whether breaks are the initiating lesion for crossovers at this fragile site, since template switching during replication can generate a double Holliday junction in the absence of a break.

Mitotic crossovers are rare in cells with wild-type levels of polymerase, but of the five events we collected in Control Diploid #1, two were at FS2 and did not have an adjacent 3∶1 gene conversion tract. The two inverted Ty1, Ty2 pairs on chromosome IV have been reported as hotspots for spontaneous crossovers [28]. Crossover events at these are usually associated with 4∶0 gene conversion, indicating an initiating lesion in G1. Although the number of spontaneous crossover events we collected is too low for a conclusive comparison with this data on inverted Ty1, Ty2 pairs from chromosome IV, FS2 likely behaves as a similar hotspot for G1-lesion stimulated crossover events in un-stressed cells.

### Comparison of gene conversion tracts associated with mitotic crossovers

In cells with low levels of polymerase, over 90% of gene conversion tracts associated with a crossover on the right arm of chromosome III are of the 3∶1 pattern, indicating an initiating lesion during S-phase. Analysis of crossovers induced by low alpha DNA polymerase on yeast chromosomes IV and V indicates that these also are associated primarily with 3∶1 rather than 4∶0 conversion tracts (W. Song and T. D. Petes, personal communication). These results are consistent with stalled or collapsed forks under replication stress stimulating crossover formation. In studies of un-stressed cells, crossover-associated gene conversion tracts on yeast chromosomes IV and V are either a mixture of 3∶1 and 4∶0, or are primarily 4∶0 [35], [39].

Our median gene conversion tract length in cells under replication stress is 14.7 kb, which is much longer than the 1–4 kb tracts observed during meiosis [57]–[59]. Other studies of mitotic crossovers in yeast have highlighted similarly extensive gene conversion with median tract lengths of 4.7 to 20.3 kb [28], [29], [35], [39], [46]. The density of SNP markers evaluated affects our ability to evaluate how often crossovers have adjacent gene conversion. In a previous report on chromosome IV where a high density of SNPs was used, 87% of crossovers had an adjacent gene conversion [28]. In our cells under replication stress, which were evaluated using fewer SNPs, only 41% of crossovers had an adjacent gene conversion.

### Summary

As discussed, we find that spontaneous mitotic BIR events on the right arm of chromosome III are more frequent than expected, compared to other yeast chromosomes. Under replication stress by low levels of polymerase alpha, the frequency of mitotic LOH is elevated approximately 12-fold, resulting from crossover, BIR, and chromosome loss. Fragile site FS2 is a hotspot for initiating LOH events under replication stress, and S-phase lesions at this site stimulate crossovers and BIR events approximately equally. More than one-third of the BIR events initiated at or near FS2 are non-allelic, resulting in gross chromosomal alteration of chromosome III. These results have important implications for adding to the mechanisms in which human common fragile sites promote tumorogenesis. Human common fragile sites, like yeast FS2, are unstable under conditions of replication stress [60] and replication fork stalling at sequences with secondary-structure forming potential has been observed in some fragile sites [6], [7]. The contribution of deletions, amplifications, and translocations at common fragile sites to tumor development and progression has been extensively documented [18], [61], [62]. However, LOH at tumor suppressor genes has long been known as a driver of tumorogenesis [63], and this mechanism has not been well studied at fragile sites. Based on our results, further research is warranted to determine the role of common fragile sites in stimulating LOH in tumors through BIR and reciprocal mitotic crossovers.

## Materials and Methods

### Strain construction

The five diploid strains used for analysis of mitotic recombination were Y332, Y382, AMC310, AMC324, and AMC331 (Figure 1). Each of these diploids was created by mating an MS71-derived haploid cell [64] with a YJM789-derived haploid cell [42], resulting in ∼0.5% sequence divergence between homologous chromosomes [42]. Each diploid is homozygous for the *ade2-1* mutation and contains one copy of *SUP4-o*. Strains Y332, Y382, AMC310, and AMC324 contain one copy of fragile site FS2; strain AMC331 does not contain any Ty1 elements at the location of FS2 on either chromosome III homolog. Strains Y332, Y382, and AMC310 are homozygous for the *GAL-POL1* construct [30]; strains AMC324 and AMC331 are homozygous for *POL1* driven under its native promoter. The configuration of genes and markers in each diploid strain is diagrammed in Figure 1. The steps in construction of these strains are described in the Text S1, and construction details and genotypes for all strains are in Tables S1 and S2. All transformations and matings were done using standard protocols.

### Genetic methods and media

All five diploid strains, whether they contained *GAL-POL1* or not, were maintained at 30°C in standard rich media [65], with the exception that the medium contained 3% raffinose instead of dextrose. Raffinose was used as a carbon source because it does not suppress the *GAL1/10* promoter, allowing us to control expression of the *GAL-POL1* construct by varying the amount of galactose in the medium.

### Induction and identification of mitotic recombination events

All diploid strains were purified to individual colonies, and were inoculated for growth overnight in standard rich media containing high galactose (0.05%). Three or four cultures were inoculated for each diploid in each condition. Cells were then washed and diluted 1∶5 in fresh rich media liquid culture with no galactose for 6 hours (to induce replication stress by lowering the level of polymerase alpha), or were diluted 1∶5 in rich media liquid culture with high galactose for 6 hours. The galactose treatment for each strain is indicated in Table 1. The density of each culture was determined, and cells were spread at low density to form colonies (∼350 colonies per plate) on plates containing high galactose and 10 µg/ml adenine (two-fold less than standard omission medium). Twenty to forty plates were spread from each culture, to obtain 7,000 to 14,000 colonies per culture. Cells were allowed to grow at 30°C for 3 days and then plates were incubated overnight at 4°C to intensify red color development in the colonies. The total number of colonies was counted for each culture, and culture counts from each diploid were totaled. If a crossover, BIR event, local gene conversion at *SUP4-o*, or chromosome loss event occurs in the first or second division at the time the diploid is plated, a sectored colony is produced. Therefore, each sectored colony is an independent event. All red/white and red/light pink sectored colonies in which the red portion was at least one-fourth of the colony were identified, and a single cell from each half of the sector was purified for subsequent analysis of the mitotic event that resulted in sectoring. The frequency of BIR events and of chromosome loss events reported in Table 1 was calculated as [number of sectored colonies of the event type/total colonies]. The frequency of crossovers reported in Table 1 was calculated for each strain as [2*number of crossover sectored colonies]/[total number of colonies].

### Statistical analysis

95% confidence intervals for the proportion [66] of each mitotic event were calculated using VassarStats (http://vassarstats.net/). Chi-square contingency tables were used to compare the frequencies of mitotic events between strains.

### Analysis and mapping of mitotic events

Phenotype analysis was initially used to classify sectored colonies. Sectors from Y332, Y382, AMC324, or AMC331 with phenotype His^+^ Hyg^S^ (red cell) and His^+^ Hyg^R^ (light pink cell) usually result from chromosome loss. Sectors from AMC310 of phenotype His^−^ Hyg^R^ (red cell) and His^+^ Hyg^R^ (light pink cell) are usually chromosome loss. Sectors from all strains in which cells from both sides of the sectored colony remain His^+^ Hyg^R^ are crossover, BIR, local gene conversion at the *SUP4-o* locus, or mutation at the *SUP4-o* locus.

There is ∼0.5% sequence divergence between the two homologs of chromosome III in the experimental diploids (Wei *et al.* 2007), and several of the single nucleotide polymorphisms (SNPs) alter restriction enzyme sites. For example, a SNP on chromosome III at base 266045 results in an *Hpy*CH4III site on the YJM789-derived chromosome but not the MS71-derived chromosome. This region was amplified by PCR, generating a 374 bp product (Table S3). If the site is heterozygous in the cell being examined, digestion of the amplified product with *Hpy*CH4III followed by gel electrophoresis reveals three band sizes: the uncut 374 bp product and the cut 259 bp and 115 bp products. Genotype analysis of the SNP at chromosome III base 266045, which is 7 kb centromere-proximal of *SUP4-o*, was used for initial evaluation of event type in all sectored colonies. In chromosome loss and BIR events, the red cell of a sector has only the form of the SNP from the copy of chromosome III lacking *SUP4-o*, and the light pink cell is heterozygous at this site. In crossover events, the red cell of a sector has only the form of the SNP from the copy of chromosome III lacking *SUP4-o*, and the white cell has only the form of the SNP from the copy of chromosome III containing *SUP4-o*. Sectors with red cells that remained homozygous at SNP 266045 may result from a point mutation in *SUP4-o* or a small gene conversion tract surrounding *SUP4-o*; these were not further analyzed.

All sectored colonies with a change of zygosity at SNP 266405 were further analyzed. Genomic DNA was harvested from purified cells from each side of sectored colonies and subjected to polymorphism analysis. For all sectored colonies, an initial set of 8 SNPs were tested for zygosity to reconfirm the event type. BIR and crossover events were then tested with additional SNPs to further refine the location of the event. In total, we used 25 SNPs in the 159 kb interval between *CEN3* and *SUP4*-*o* on chromosome III, plus 2 additional SNPs centromere-distal from *SUP4-o*. Polymorphic sites, primers, and diagnostic restriction enzymes are listed in Table S3. Gene conversion tract lengths were calculated as described in Lee *et al.* (2009) with the modification that the size of Ty1 elements present in the MS71-derived homolog that are not present in the sequenced genome are accounted for in our distance calculations when this homolog is used as the template for repair.

### Analysis of BIR events by pulsed-field gel electrophoresis (PFGE) and Southern blotting

Genomic DNA from 1×10^8^ cells was harvested in agarose blocks to prevent shearing as described in [67]. Chromosomes were separated by PFGE in a 1.2% gel in 0.5× TBE at 14°C using a Gene Navigator system (Pharmacia Biotech). Switch times at 6 V/cm were as follows: 50 sec switch for 4.5 hr, 90 sec switch for 5.5 hr, 105 sec switch for 7.5 hr, 124 sec switch for 7.5 hr, 170 sec switch for 7.5 hr. DNA was transferred to Hybond N+ membrane (GE Healthcare Life Sciences) by a neutral transfer according to standard protocol, then probed with *CHA1*, a gene located on the left arm of chromosome III. The *CHA1* probe was made by PCR, using primers 5′ CTGGAAATATGAAATTGTCAGCGAC and 5′ TGAATGCCTTCAACCAAGTGGCCCTTTC. Probes were radioactively labeled by random-prime labeling using Ready To Go beads (-dCTP) (GE Healthcare Life Sciences). Southern blot hybridization and washes were standard. Membranes were exposed to a phosphor screen and images were captured with an FLA-3000 scanner (Fujifilm). There are two normal sizes for chromosome III in our diploids; the YJM789-derived homolog of this chromosome is ∼18 kb smaller than the MS71-derived homolog, because the YJM789 homolog has only one Ty1 element and the MS71 homolog has four Ty1 elements on the right arm of chromosome III. Diploids with two normal-size copies of chromosome III were considered allelic BIR events. Diploids with one normal-size III and one chromosome III of abnormal size were considered non-allelic BIR events.

## Supporting Information

Figure S1 Allelic and non-allelic BIR events. A subset of 35 BIR events from Experimental Diploid #1 under replication stress were evaluated. The diagram and color format is the same as described in Figure 6. BIR events that were evaluated by CHEF and Southern blotting with a *CHA1* probe to the right arm of chromosome III are shown by arrowheads below the chromosomes. Solid red arrowheads indicate that both copies of chromosome III are of normal size thus the BIR event is allelic. Arrowheads with a yellow center indicate that one chromosome III is of abnormal size, thus the BIR event is non-allelic.
(TIF)

Figure S2 BIR events with associated gene conversion. SNP testing results from three sectored colonies, SC100, SC104, and SC121 are shown. In each sectored colony, results from the red side of the sector are shown at the top of each diagram, and results from the white side of the sector are shown at the bottom of each diagram. The two homologs of the right arm of chromosome III are shown. Large ovals represent the centromere, and centromeres are labeled to indicate the MS71-derived YJM789-derived homologs. SNP markers used to map events are shown by circles on the chromosome diagrams. Numbers are the approximate chromosome coordinate in kb. A red circle indicates the MS71 form of the SNP is present, and a black circle indicates the YJM789 form of the SNP is present.
(TIF)

Table S1 Strain list and strain constructions for MS71-derived haploids.
(DOC)

Table S2 Strain list and strain constructions for YJM789-derived haploids.
(DOC)

Table S3 Names and sequences of primers used to test SNPs on the right arm of chromosome III.
(DOC)

Text S1 Supplemental materials and methods on construction of Experimental Diploids #1 and 2 and Control Diploids #1, 2, and 3.
(DOCX)
