# Supplementary figures and images for: Fragile Site Instability in Saccharomyces cerevisiae Causes Loss of Heterozygosity by Mitotic Crossovers and Break-Induced Replication
Source: PLoS Genet. 2013 Sep 19;9(9):e1003817. doi: 10.1371/journal.pgen.1003817 (PMC3778018; doi:10.1371/journal.pgen.1003817)

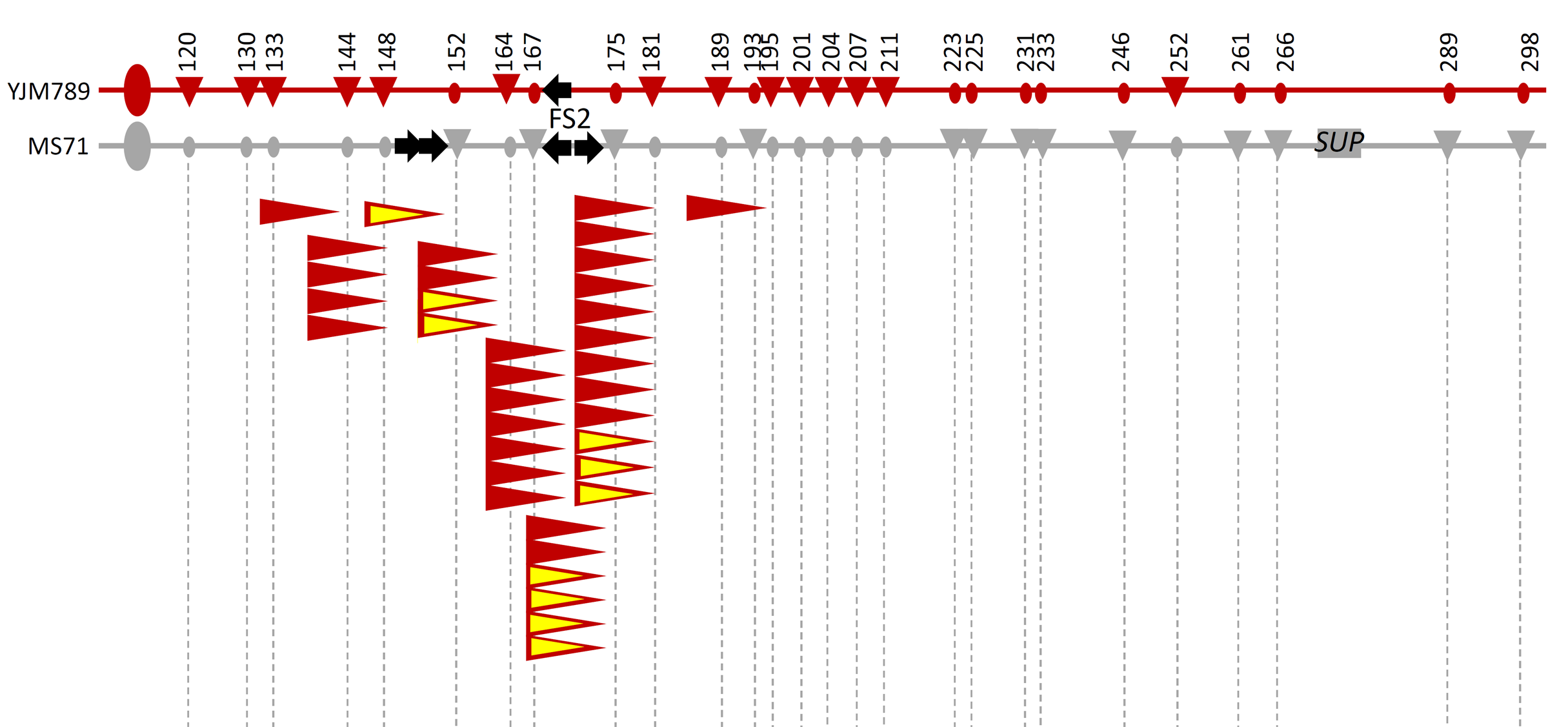

Supplement: Figure S1 — Allelic and non-allelic BIR events. A subset of 35 BIR events from Experimental Diploid #1 under replication stress were evaluated. The diagram and color format is the same as described in Figure 6. BIR events that were evaluated by CHEF and Southern blotting with a CHA1 probe to the right arm of chromosome III are shown by arrowheads below the chromosomes. Solid red arrowheads indicate that both copies of chromosome III are of normal size thus the BIR event is allelic. Arrowheads with a yellow center indicate that one chromosome III is of abnormal size, thus the BIR event is non-allelic. (TIF) [file pgen.1003817.s001.tif]

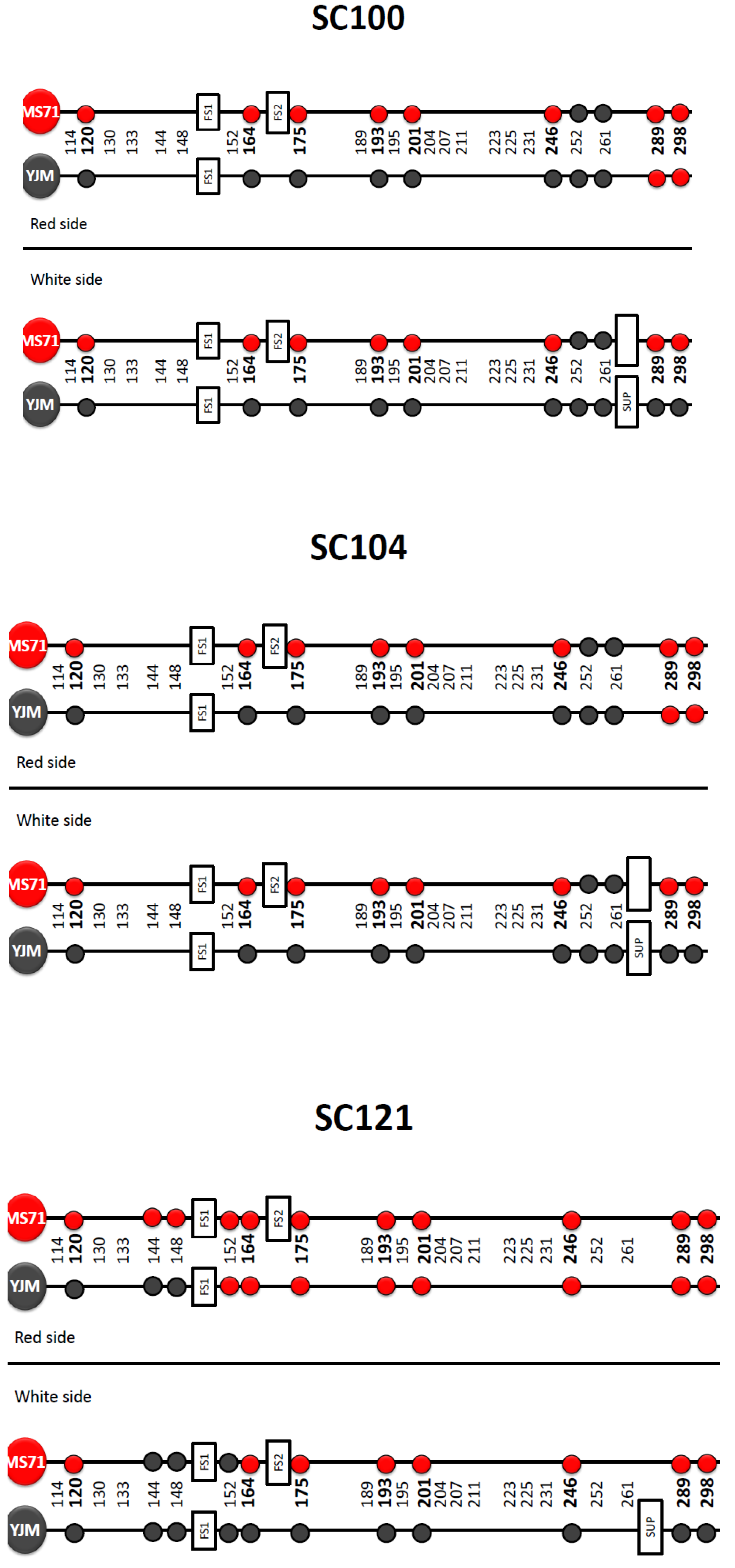

Supplement: Figure S2 — BIR events with associated gene conversion. SNP testing results from three sectored colonies, SC100, SC104, and SC121 are shown. In each sectored colony, results from the red side of the sector are shown at the top of each diagram, and results from the white side of the sector are shown at the bottom of each diagram. The two homologs of the right arm of chromosome III are shown. Large ovals represent the centromere, and centromeres are labeled to indicate the MS71-derived YJM789-derived homologs. SNP markers used to map events are shown by circles on the chromosome diagrams. Numbers are the approximate chromosome coordinate in kb. A red circle indicates the MS71 form of the SNP is present, and a black circle indicates the YJM789 form of the SNP is present. (TIF) [file pgen.1003817.s002.tif]
