# Supplementary material for: Fragile Site Instability in Saccharomyces cerevisiae Causes Loss of Heterozygosity by Mitotic Crossovers and Break-Induced Replication
Source: PLoS Genet. 2013 Sep 19;9(9):e1003817. doi: 10.1371/journal.pgen.1003817 (PMC3778018; doi:10.1371/journal.pgen.1003817)
Supplement: Table S1 — Strain list and strain constructions for MS71-derived haploids. (DOC) [file pgen.1003817.s003.doc]

**TABLE S1**

**Strain genotypes and constructions for MS71-derived haploids**

| **Strain name** | **Relevant genotype*^a^*** | **Reference or construction*^b^*** |
| --- | --- | --- |
| EAS18 | *MAT*a | Lemoine 2005 |
| AMC207 | *can1::*pCORE | Transformation of EAS18 with *Kan*MX4*-URA3* cassette, pCORE template [1]; primers AMC228 (5’AGAATGCGAAATGGCGTGGAAATGTGATCAAAGGTAATAAAACGTCATATtccttaccattaagttgatc) and AMC229 (5’CAATCGAAAGTTTATTTCAGAGTTCTTCAGACTTCTTAACTCCTGTAAAAgagctcgttttcgacactgg) |
| AMC208 | *can1*Δ | Transformation of AMC207 with integrative recombinant oligonucleotides AMC230 (5’ATGGCGTGGAAATGTGATCAAAGGTAATAAAACGTCATATTTTTACAGGAGTTAAGAAGTCTGAAGAACTCTGAAATAAA) and AMC231 (5’TTTATTTCAGAGTTCTTCAGACTTCTTAACTCCTGTAAAAATATGACGTTTTATTACCTTTGATCACATTTCCACGCCAT) |
| AMC211 | *can1*Δ *ade5-1::*pCORE | Transformation of AMC208 with *Kan*MX4*-URA3* cassette, pCORE template [1]; primers AMC258 (5’ACATACGCAGACTCAGGTGTCTCTGTTGATAATGGTAACAATCTCGTACAgagctcgttttcgacactgg) and AMC259 (5’CAGAGTCTGCACCTGGCCTTCTTGTGGATCTGACCATTTCTTTGATAGTTtccttaccattaagttgatc) |
| AMC215 | *can1*Δ *ADE5* | Transformation of AMC211 with *ADE5* allele, genomic DNA template from strain W303 [2]; primers AMC247 (5’ACCGTCCCAGGAGCTACCAC) and AMC249 (5’CGCTCGTTAAAGGTATTAATTGTCC) |
| AMC274 | *can1*Δ *ADE5* III273292::pCORE | Transformation of AMC215 with *Kan*MX4*-URA3* cassette, pCORE template [1]; primers AMC392 (5’AAACACGTCAGTTCCTTCTGTCTGTTGTAAATAGGATGCATCCGCAGTGAtccttaccattaagttgatc) and AMC393 (5’TACGGAGCGTTTATGGCTTCATTGACCAAATAAAATGAT TGGGCTGAAAGgagctcgttttcgacactgg) |
| AMC281 | *can1*Δ *ADE5* III273292::*can1-100* | Transformation of AMC274 with *can1-100* allele, genomic DNA template from strain PSL2 [3]; primers AMC398 (5’AAACACGTCAGTTCCTTCTGTCTGTTGTAAATAGGATGC ATCCGCAGTGAAgggtgagaatgcgaaatgg) and AMC399 (5’TACGGAGCGTTTATGGCTTCATTGACCAAATAAAAT GATTGGGCTGAAAGgtgtggtttccgggtgagtc) |
| AMC285 | *can1*Δ *ADE5* III273292::*can1-100 his4::HPH* | Transformation of AMC281 with *his4::HPH*; pAG32 template [4]; primers AMC265 (5’ TGGAGAACTGGAGAATCTCTTCATTACTCAGGCTCGAGCCATCCAAAAGTatcgatgaattcgagctcg) and AMC266 (5’ CTGGCCTCATGGAATAGTAAGAAGGAATACGTTTCACTTGTTGGTCAGGTcgtacgctgcaggtcgac) |
| AMC293 | *can1*Δ *ADE5* III273292::*can1-100 his4::HPH* *ade2*::pCORE | Transformation of AMC285 with *Kan*MX4*-URA3* cassette, pCORE template [1]; primers AMC382 (5’ GGGACGTCTC ACTGGCTTGT TCCACAGGAA CACTTTGGGT AACTGCTATA tccttaccattaagttgatc) and AMC383 (5’ TCCTGCCAAA CAAATAAGCA ACTCCAATGA CCACGTTAAT GGCTCCTTTT gagctcgttttcgacactgg) |
| AMC296 | *can1*Δ *ADE5* III273292::*can1-100 his4::HPH* *ade2*-1 | Transformation of AMC293 with *ade2-1* allele, genomic DNA template from strain PSL2 [3]; primers AMC384 (5’ GCAGGCGCATAACATAAGTC) and AMC385 (5’ ACTCTTGTTGCATGGCTACG) |
| AMC304 | *can1*Δ *ADE5* III273292::*can1-100 his4::HPH* *ade2*-1 III313553::*URA3* | Transformation of AMC296 with *URA3*  allele, pCORE template [1]; primers AMC405 (5’GAAACACTGTTATCCTCCACGTTTTTTCCACTGTTTTAAGACTCGACAAG gttacctcactcattagg) and AMC406 (5’TTGACGAGCAGGTTAAAAGAAACACTGCGAAATGTGAGGAATCTTTCCGTgttgaagtgagtgttgcac) |
| AMC306 | *can1*Δ *ADE5* III273292::*can1-100 his4::HPH* *ade2*-1 III313553::*URA3 GAL-POL1* | Transformation of AMC304 with *GAL-POL1* allele, genomic DNA template from strain NPD1 [5]; primers P60 (5’ TTTCTTGTACTGCCTGCAATCTC) and P61 (5’CATTTGCGTAGCGCAGTTTC) |
| Y261 | *can1*Δ *ADE5* III273292::*can1-100 his4::HPH* *ade2*-1 III273292::pCORE | Transformation of AMC296 with *Kan*MX4*-URA3* cassette, pCORE template [1]; primers AMC392 (5’AAACACGTCAGTTCCTTCTGTCTGTTGTAAATAGGATGCATCCGCAGTGAtccttaccattaagttgatc) and AMC393 (5’TACGGAGCGTTTATGGCTTCATTGACCAAATAAAATGAT TGGGCTGAAAGgagctcgttttcgacactgg) |
| Y317 | *can1*Δ *ADE5* III273292::*can1-100 his4::HPH* *ade2*-1 III273292::*SUP4*-o | Transformation of Y261 with *SUP4-*o allele, genomic DNA template from strain PSL5 [3]; primers AMC400 (5’AAACACGTCAGTTCCTTCTGTCTGTTGTAAATAGGATGCATCCGCAGTGAggatccggaattcttgaaag) and AMC401 (5’TACGGAGCGTTTATGGCTTCATTGACCAAATAAAATGATTGGGCTGAAAGggatccgggaccggataat) |
| Y328 | *can1*Δ *ADE5* III273292::*can1-100 his4::HPH* *ade2*-1 III273292::*SUP4*-o *GAL-POL1* | Transformation of Y317 with *GAL-POL1* allele, genomic DNA template from strain NPD1 [5]; primers P60 (5’TTTCTTGTACTGCCTGCAATCTC) and P61 (5’CATTTGCGTAGCGCAGTTTC) |
| Y368 | *can1*Δ *ADE5* III273292::*can1-100 his4::HPH* *ade2*-1 III273292::*SUP4*-o *GAL-POL1* III169125::*NAT* | Transformation of Y328 with III169125::*NAT*; pAG25 template [4]; primers P240 (5’ TTCTAGATACCGACATATCAATATAAAAATACTGAATATTGAGACATCTTcgtacgctgcaggtcgac) and P241 (5’ CACCCATTTAAATTGCATTTTTGGTTTGCGGCAACCAAAACGTAATCTAAatcgatgaattcgagctcg) |
| AMC330 | *can1*Δ *ADE5* III273292::*can1-100 his4::HPH* *ade2*-1 III273292::*SUP4*-o *fs2* Δ*::NAT* | Transformation of Y317 with III168239::*NAT*; pAG25 template [4]; primers AMC451 (5’ AAACCTGGCAGAAGCGTCTTGTTAATACTTATAGAGAAACCACCAGTAGCcgtacgctgcaggtcgac) and AMC452 (5’ TTTCTCTTAGCTCGATTTATTTACCTTTATTTAACTTCTG CAGTTGGACAatcgatgaattcgagctcg) |

*^a^* All strains are isogenic with MS71, a *LEU2* derivative of AMY125 (*α ade5-1 leu2-3 trp1-289 ura3-52 his7-2*) [6] except for changes introduced as noted under “Relevant Genotype”.

*^b^* All genetic manipulations (transformations, matings, and tetrad dissections) were done using standard protocols. For strains constructed by transformation using PCR fragments to the targeted location, both the template for PCR amplification and primers are indicated. Primer sequences are shown with upper case letters corresponding to the targeted genomic regions and lower case letters corresponding to the selectable marker.

**REFERENCES**

1. Storici F, Lewis LK, Resnick MA (2001) In vivo site-directed mutagenesis using oligonucleotides. Nat Biotechnol 19: 773-776.

2. Winzeler EA, Castillo-Davis CI, Oshiro G, Liang D, Richards DR, et al. (2003) Genetic diversity in yeast assessed with whole-genome oligonucleotide arrays. Genetics 163: 79-89.

3. Lee PS, Greenwell PW, Dominska M, Gawel M, Hamilton M, et al. (2009) A fine-structure map of spontaneous mitotic crossovers in the yeast Saccharomyces cerevisiae. PLoS Genet 5: e1000410.

4. Goldstein AL, McCusker JH (1999) Three new dominant drug resistance cassettes for gene disruption in Saccharomyces cerevisiae. Yeast 15: 1541-1553.

5. Lemoine FJ, Degtyareva NP, Lobachev K, Petes TD (2005) Chromosomal translocations in yeast induced by low levels of DNA polymerase a model for chromosome fragile sites. Cell 120: 587-598.

6. Kokoska RJ, Stefanovic L, DeMai J, Petes TD (2000) Increased rates of genomic deletions generated by mutations in the yeast gene encoding DNA polymerase delta or by decreases in the cellular levels of DNA polymerase delta. Mol Cell Biol 20: 7490-7504.
