# Supplementary material for: Fragile Site Instability in Saccharomyces cerevisiae Causes Loss of Heterozygosity by Mitotic Crossovers and Break-Induced Replication
Source: PLoS Genet. 2013 Sep 19;9(9):e1003817. doi: 10.1371/journal.pgen.1003817 (PMC3778018; doi:10.1371/journal.pgen.1003817)
Supplement: Table S2 — Strain list and strain constructions for YJM789-derived haploids. (DOC) [file pgen.1003817.s004.doc]

**TABLE S2**

**Strain genotypes and constructions for YJM789-derived haploids**

| **Strain name** | **Relevant genotype*^a^*** | **Reference or construction*^b^*** |
| --- | --- | --- |
| YJM789 |  | [1] |
| AMC231 | *ura3* | 5-FOA resistant clone of YJM789 |
| AMC236 | *ura3 trp1::*pCORE | Transformation of AMC231 with *Kan*MX4*-URA3* pCORE cassette, pCORE template [2]; primers AMC222 (5’TATTGAGCACGTGAGTATACGTGATTAAGCACACAAAGGCAGCTTGGAGTgagctcgttttcgacactgg) and AMC260 (5’ATTTGTCTCCACACCTCCGCTTACATCAACACCAATAACGCCATTTAATCtccttaccattaagttgatc) |
| AMC239 | *ura3 trp1*Δ | Transformation of AMC236 with integrative recombinant oligonucleotides AMC224 (5’GTGAGTATACGTGATTAAGCACACAAAGGCAGCTTGGAGTGTTATTACTGAGTAGTATTTATTTAAGTATTGTTTGTGCA) and AMC225 (5’TGCACAAACAATACTTAAATAAATACTACTCAGTAATAACACTCCAAGCTGCCTTTGTGTGCTTAATCACGTATACTCAC) |
| AMC247 | *ura3 trp1*Δ III168239::pCORE | Transformation of AMC239 with *Kan*MX4*-URA3* pCORE cassette, pCORE template [2]; primers AMC222 (5’TATTGAGCACGTGAGTATACGTGATTAAGCACACAAAGGCAGCTTGGAGTgagctcgttttcgacactgg) and AMC260 (5’ATTTGTCTCCACACCTCCGCTTACATCAACACCAATAACGCCATTTAATCtccttaccattaagttgatc) |
| AMC255 | *ura3 trp1*Δ III168239::*Ty1* | Transformation of AMC247 with Crick-orientation *Ty1* gene, genomic DNA template from strain MS71 [3]; primers 27C-1 (5’ctctgaaagttatgtgtggagttc) and RFS2u#2 (5’cacccatttaaattgcatttttg) |
| AMC260 | *ura3 trp1*Δ/III313553::*TRP1* III168239::*Ty1* | Transformation of AMC255 with *TRP1* allele, genomic DNA template from strain PSL5 [4]; primers AMC359 (5’GAAACACTGTTATCCTCCACGTTTTTTCCACTGTTTTAAGACTCGACAAGtgaatgaacgtatacgcg) and AMC378 (5’TTGACGAGCAGGTTAAAAGAAACACTGCGAAATGTGAGGAATCTTTCCGTcagtaataacctatttcttagc) |
| AMC264 | *ura3 trp1*Δ/III313553::*TRP1* III168239::*Ty1 can1::*pCORE | Transformation of AMC260 with *Kan*MX4*-URA3* pCORE cassette, pCORE template [2]; primers AMC228 (5’ AGAATGCGAAATGGCGTGGAAATGTGATCAAAGGTAATAAAACGTCATATtccttaccattaagttgatc) and AMC229 (5’ CAATCGAAAGTTTATTTCAGAGTTCTTCAGACTTCTTAACTCCTGTAAAAgagctcgttttcgacactgg) |
| AMC266 | *ura3 trp1*Δ/III313553::*TRP1* III168239::*Ty1 can1*Δ | Transformation of AMC264 with integrative recombinant oligonucleotides AMC230 (5’ATGGCGTGGAAATGTGATCAAAGGTAATAAAACGTCATATTTTTACAGGAGTTAAGAAGTCTGAAGAACTCTGAAATAAA) and AMC231 (5’TTTATTTCAGAGTTCTTCAGACTTCTTAACTCCTGTAAAAATATGACGTTTTATTACCTTTGATCACATTTCCACGCCAT) |
| AMC269 | *ura3 trp1*Δ/III313553::*TRP1* III168239::*Ty1 can1*Δ *ade2::*pCORE | Transformation of AMC266 with *Kan*MX4*-URA3* pCORE cassette, pCORE template [2]; primers AMC382 (5’GGGACGTCTCACTGGCTTGTTCCACAGGAACACTTTGGGTAACTGCTATAtccttaccattaagttgatc) and AMC383 (5’TCCTGCCAAACAAATAAGCAACTCCAATGACCACGTTAATGGCTCCTTTTgagctcgttttcgacactgg) |
| AMC273 | *ura3 trp1*Δ/III313553::*TRP1* III168239::*Ty1 can1*Δ *ade2-1* | Transformation of AMC269 with *ade2-1* allele, genomic DNA template from strain PSL2 [4]; primers AMC384 (5’GCAGGCGCATAACATAAGTC) and AMC385 (5’ACTCTTGTTGCATGGCTACG) |
| AMC298 | *ura3 trp1*Δ/III313553::*TRP1* III168239::*Ty1 can1*Δ *ade2-1* III273292::pCORE | Transformation of AMC273 with *Kan*MX4*-URA3* pCORE cassette, pCORE template [2]; primers AMC392 (5’ AAACACGTCAGTTCCTTCTGTCTGTTGTAAATAGGATGCATCCGCAGTGAtccttaccattaagttgatc) and AMC393 (5’TACGGAGCGTTTATGGCTTCATTGACCAAATAAAATGATTGGGCTGAAAGgagctcgttttcgacactgg) |
| AMC302 | *ura3 trp1*Δ/III313553::*TRP1* III168239::*Ty1 can1*Δ *ade2-1* III273292::*SUP4*-o | Transformation of AMC298 with *SUP4-*o allele, genomic DNA template from strain PSL5 [4]; primers AMC400 (5’AAACACGTCAGTTCCTTCTGTCTGTTGTAAATAGGATGCATCCGCAGTGAggatccggaattcttgaaag) and AMC401 (5’TACGGAGCGTTTATGGCTTCATTGACCAAATAAAATGATTGGGCTGAAAGggatccgggaccggataat) |
| AMC308 | *ura3 trp1*Δ/III313553::*TRP1* III168239::*Ty1 can1*Δ *ade2-1* III273292::*SUP4*-o *GAL-POL1* | Transformation of AMC298 with *GAL-POL1* allele, genomic DNA template from strain NPD1 [3]; primers P60 (5’TTTCTTGTACTGCCTGCAATCTC) and P61 (5’CATTTGCGTAGCGCAGTTTC) |
| Y325 | *ura3 trp1*Δ/III313553::*TRP1* III168239::*Ty1 can1*Δ *ade2-1 GAL-POL1* | Transformation of AMC273 with *GAL-POL1* allele, genomic DNA template from strain NPD1 [3]; primers P60 (5’TTTCTTGTACTGCCTGCAATCTC) and P61 (5’CATTTGCGTAGCGCAGTTTC) |
| AMC328 | *ura3 trp1*Δ/III313553::*TRP1* III168239::*Ty1 can1*Δ *ade2-1 fs2* Δ*::NAT* | Transformation of AMC273 with III168239::*NAT*; pAG25 template [5]; primers AMC451 (5’ AAACCTGGCAGAAGCGTCTTGTTAATACTTATAGAGAAACCACCAGTAGCcgtacgctgcaggtcgac) and AMC452 (5’ TTTCTCTTAGCTCGATTTATTTACCTTTATTTAACTTCTG CAGTTGGACAatcgatgaattcgagctcg) |

*^a^* All strains are isogenic with YJM789 (*MAT*α *ho::hisG lys2 gal2*) (Wei W 2007) except for changes introduced as noted under “Relevant Genotype”.

*^b^* All genetic manipulations (transformations, matings, and tetrad dissections) were done using standard protocols. For strains constructed by transformation using PCR fragments to the targeted location, both the template for PCR amplification and primers are indicated. Primer sequences are shown with upper case letters corresponding to the targeted genomic regions and lower case letters corresponding to the selectable marker on the plasmid.

**REFERENCES**

1. Wei W, McCusker JH, Hyman RW, Jones T, Ning Y, et al. (2007) Genome sequencing and comparative analysis of Saccharomyces cerevisiae strain YJM789. Proc Natl Acad Sci U S A 104: 12825-12830.

2. Storici F, Lewis LK, Resnick MA (2001) In vivo site-directed mutagenesis using oligonucleotides. Nat Biotechnol 19: 773-776.

3. Lemoine FJ, Degtyareva NP, Lobachev K, Petes TD (2005) Chromosomal translocations in yeast induced by low levels of DNA polymerase a model for chromosome fragile sites. Cell 120: 587-598.

4. Lee PS, Greenwell PW, Dominska M, Gawel M, Hamilton M, et al. (2009) A fine-structure map of spontaneous mitotic crossovers in the yeast Saccharomyces cerevisiae. PLoS Genet 5: e1000410.

5. Goldstein AL, McCusker JH (1999) Three new dominant drug resistance cassettes for gene disruption in Saccharomyces cerevisiae. Yeast 15: 1541-1553.
