# Supplementary material for: Fragile Site Instability in Saccharomyces cerevisiae Causes Loss of Heterozygosity by Mitotic Crossovers and Break-Induced Replication
Source: PLoS Genet. 2013 Sep 19;9(9):e1003817. doi: 10.1371/journal.pgen.1003817 (PMC3778018; doi:10.1371/journal.pgen.1003817)
Supplement: Table S3 — Names and sequences of primers used to test SNPs on the right arm of chromosome III. (DOC) [file pgen.1003817.s005.doc]

**TABLE S3**

**Primers used to test SNPs**

| **Primer Sequence, 5’ to 3’** | | **Comments** |
| --- | --- | --- |
| AGCCGGCAAATTACCAAACC | CACTATTCCGGCAGCTAGTTGATAC | Generates 452 bp product around polymorphic 120340; cuts in SGD with *Hinf*I (282, 170 fragments) |
| CCGTTCCGTACAGAAGAATTAACG | ATAATAACCGCGGGCATGTG | Generates 422 bp product around polymorphic 130370; cuts in SGD with *Msp*I (236, 186 fragments) |
| ACTCAGCCGCTCGCCTATTC | CAAACGTGGCCTTCAAGTACC | Generates 395 bp product around polymorphic 133713; cuts in SGD with *Bsa*HI (274, 121 fragments) |
| CCTGATCAAAGACGAGCCATC | TGCTCCGAGTCATCATTCTTCC | Generates 441 bp product around polymorphic 144295; cuts in SGD with *Nhe*I (265, 175 fragments) |
| GTAGAAGTTCACTTAAACTTATTGAGC | TTTGTAGTCCCTGTGGACTCG | Generates 370 bp product around polymorphic 148113; cuts in SGD with *Rsa*I (223, 147 fragments) |
| CTGAAATGAAGAGGAATGACG | GCTGAGGATCTCGGAAAC | Generates 352 bp product around polymorphic 152544; cuts in YJM789 with *Alu*I (238, 114 fragments) |
| TCGACTAAATCGTCGATGCTG | CCAATGATGGGACTGGGATG | Generates 442 bp product around polymorphic 164273; cuts in YJM789 with *Msp*I (288, 154 fragments) |
| CCAGAGTTCCTTCGACACG | AAATTCTAGATACTGCAGGCCAAG | Generates 390 bp product around polymorphic 167720; cuts in SGD with *Hpy*CH4III (311, 79 fragments) |
| GGCAGTAACAGGTGCATCTACG | AAAGTCGTCCCACACCCTAAG | Generates 298 bp product around polymorphic 175324; cuts in YJM789 with *Ban*I (192, 106 fragments) |
| CTGGCGTCAGGCGGATTAG | TTCCACTATTTGATCCATAATCATCAG | Generates 438 bp product around polymorphic 181520; cuts in SGD with *Bts*I (257, 181 fragments) |
| TTCAGCCGGTTCTAGTTCAAGG | CCATTGAGAGCCAAACTCCTTC | Generates 398 bp product around polymorphic 189048; cuts in SGD with *Alu*I (229, 169 fragments) |
| TCACGTCGGTACTTACGAAGG | CTTTCCACGATTTGAAGACC | Generates 351 bp product around polymorphic 193671; cuts in YJM789 with *Hinf*I (229, 122 fragments) |
| GGGAGGCAACGTTGGAAATC | CACCGGTGTATGGCATTGG | Generates 364 bp product around polymorphic 195583; cuts in SGD with *Alu*I (215, 149 fragments) |
| TGCTGAAGTACGTGGTGACG | GAACCGCATGGGCAGTTTAC | Generates 428 bp product around polymorphic 201157; cuts in SGD with *Hinf*I (250, 178 fragments) |
| CCTCAAACTCGGATTGTTGC | CAAGCCCTGGTTATGGAACG | Generates 421 bp product around polymorphic 204526; cuts in SGD with *Hinf*I (264, 157 fragments) |
| ACTGACGCAGTGAACGCTTG | CAGGAATTCTTCTCGACGATATTC | Generates 332 bp product around polymorphic 207530; cuts in SGD with *Hinf*I (255, 77 fragments) |
| TGGTCGAACCCGTGTACTTG | TGAACAACCACCTGCGTGAG | Generates 423 bp product around polymorphic 211768; cuts in SGD with *Hinf*I (306, 117 fragments) |
| TAAACCGGTCGGGACCTATG | AGACAGAACCGGTCCAGCAG | Generates 369 bp product around polymorphic 223672; cuts in YJM789 with *Dpn*II (202, 167 fragments) |
| TTGGCAACTAACGGAACTAAGG | CCGCGCAGCCGAATAAAC | Generates 446 bp product around polymorphic 225410; cuts in YJM789 with *Hae*III (255, 191 fragments) |
| CCCGTCTTAACACCTTGTGG | CATGGATCTTTTGGCAAGTTTTC | Generates 351 bp product around polymorphic 231854; cuts in SGD with *Nla*III (248, 103 fragments) |
| CCCTTCAATATTTGTGGATTCTG | TGCTTCTGAATCAATCCCTTC | Generates 369bp product around polymorphic 233758; cuts in SGD with *Hpy*CH4III (95, 274 fragments) |
| CGTATCGGTCCTTAAAAGTCAGC | TTTCTCTTGCCTTGGTACCTTATG | Generates 355 bp product around polymorphic 246475; cuts in YJM789 with *Hinf*I (233, 100 fragments) |
| TTGCTCTTCAGGCGACAAATC | ACAGCCAATCTTGTCGATGC | Generates 364 bp product around polymorphic 252005; cuts in SGD with *Hinf*I (207, 157 fragments) |
| GAGCATCGGGAACAGACTGG | CCACTTTGCCTGTGGTGTCC | Generates 429 bp product around polymorphic 261761; cuts in YJM789 with *Rsa*I (252, 177 fragments) |
| GGCTAAGGAGGACCCACGTC | CTGAAGCGGCCAATCCTTC | Generates 374 bp product around polymorphic 266045; cuts in YJM789 with *Hpy*CH4III (259, 115 fragments) |
| CCGCATACGGTAAGGACAGC | GCAGTTGTTGCTGCTCAAACG | Generates 466 bp product around polymorphic 289633; cuts in YJM789 with *Msp*I (284, 182 fragments) |
| ACGTCTGCGGCTGGTTGAC | CCTACGGTCTTCCGCGTTG | Generates 353 bp product around polymorphic 298875; cuts in YJM789 with *Rsa*I (226, 127 fragments) |
