# Supplementary material for: Fragile Site Instability in Saccharomyces cerevisiae Causes Loss of Heterozygosity by Mitotic Crossovers and Break-Induced Replication
Source: PLoS Genet. 2013 Sep 19;9(9):e1003817. doi: 10.1371/journal.pgen.1003817 (PMC3778018; doi:10.1371/journal.pgen.1003817)
Supplement: Text S1 — Supplemental materials and methods on construction of Experimental Diploids #1 and 2 and Control Diploids #1, 2, and 3. (DOCX) [file pgen.1003817.s006.docx]

**Supplemental Text**

**Strain construction:** Diploid AMC324 results from a cross between haploid AMC273 and haploid Y317. Diploid Y332 results from a cross between haploid Y325 and haploid Y328. Diploid AMC310 results from a cross between haploid AMC308 and haploid AMC306. Diploid Y382 results from a cross between haploid Y325 and haploid Y368. Diploid AMC331 results from a cross between haploid AMC328 and haploid AMC330. The construction of each of these haploids is described below, and construction details and genotypes for all strains are in Supplemental Tables 1 and 2.

Haploid AMC273 is isogenic with strain YJM789 (*MAT*α *ho::hisG lys2 gal2*) (Wei *et al.* 2007) and was created in the following steps. Initially, strain YJM789 was plated on medium containing 5-FOA to identify cells with a mutation in *ura3*. A 5-FOA resistant, *ura3* mutant colony was chosen and purified, creating strain AMC231. The *TRP1* gene was then replaced with the *Kan*MX4-*URA3* cassette from pCORE (Storici *et al.* 2001), resulting in strain AMC236. This *Kan*MX4-*URA3* cassette was deleted by transformation with integrative recombinant oligonucleotides, creating *trp1*Δ strain AMC239. The pCORE cassette was then inserted on chromosome III at base 168239 (this is the location of the centromere-proximal Ty1 of FS2 in the MS71-derived strain), creating strain AMC247. This pCORE cassette was then replaced by a Crick-orientation Ty1 element, by transformation with a PCR product from amplification of the corresponding Ty1 element of FS2 from the MS71-derived strain, creating strain AMC255. A wild-type *TRP1* allele was then inserted at chromosome III base 313553, creating strain AMC260. The *CAN1* allele was then disrupted with the pCORE cassette, creating strain AMC264. This pCORE cassette was deleted by transformation with integrative recombinant oligonucleotides, creating *can1*Δ strain AMC266. The *ADE2* gene was then disrupted with the pCORE cassette, resulting in strain AMC269. This pCORE cassette was replaced with the *ade2-1* allele, creating strain AMC273. The *ade2-1* allele in strain AMC273 was confirmed by sequencing.

Haploid Y317 is isogenic with strain MS71 (*MAT α ade5-1 his7-2 ura3-52 trp1-289*) (Lemoine *et al.* 2005) and was created in the following steps. Starting with strain EAS18, an isogenic derivative of MS71 with the opposite mating type (*MAT*a ade5-1 his7-2 ura3-52 trp1-289) (Lemoine *et al.* 2005), the *CAN1* allele was disrupted with the *Kan*MX4-*URA3* cassette from pCORE, creating strain AMC207. This pCORE cassette was deleted by transformation with integrative recombinant oligonucleotides, creating *can1*Δ strain AMC208. The *ade5* gene was disrupted by transformation with the *Kan*MX4-*URA3* cassette from pCORE (Storici *et al.* 2001), resulting in strain AMC211. This *Kan*MX4-*URA3* cassette was replaced by transformation with the wild-type *ADE5* allele, creating strain AMC215. The wild-type *ADE5* allele in strain AMC215 was confirmed by sequencing. The pCORE cassette was then inserted on chromosome III at base 273292, creating strain AMC274. This pCORE cassette was replaced by the *can1-100* allele, creating strain AMC281. The *can1-100* allele was confirmed by sequencing. Although the sequence of this inserted allele and upstream promoter region is correct, this gene does not function as anticipated; we are unable to suppress canavanine resistance with *SUP4-o*. Therefore, although *can1-100* is present in this strain, we did not use it in our experiments. The *HIS4* gene was then replaced by transformation with the *HPH* gene for hygromycin resistance, creating strain AMC285. The *ADE2* gene was then disrupted with the pCORE cassette, resulting in strain AMC293. This pCORE cassette was replaced with the *ade2-1* allele, creating strain AMC296. The *ade2-1* allele in strain AMC296 was confirmed by sequencing. Strain AMC296 was transformed with the pCORE cassette targeted for insertion on chromosome III at base 273292, creating strain Y261. This pCORE cassette was then replaced by transformation with a PCR product containing the *SUP4*-o gene amplified from strain PSL5 (Lee *et al.* 2009), creating strain Y317.

Haploid Y325 is isogenic with strain YJM789 (Wei *et al.* 2007) and was created by transformation of strain AMC273 with a PCR product of the *GAL-POL1* construct amplified from yeast strain NPD1 (Lemoine *et al.* 2005).

Haploid Y328 is isogenic with strain MS71 (Lemoine *et al.* 2005) and was created by transformation of strain Y317 with a PCR product of the *GAL-POL1* construct amplified from yeast strain NPD1 (Lemoine *et al.* 2005).

Haploid AMC308 is isogenic with strain YJM789 (Wei *et al.* 2007) and was created in the following steps. Strain AMC273 was transformed with the pCORE (Storici *et al.* 2001) cassette targeted to chromosome III at base 273292, creating strain AMC298. This pCORE cassette was then replaced by transformation with a PCR product containing the *SUP4*-o gene amplified from strain PSL5 (Lee *et al.* 2009), creating strain AMC302. Finally, transformation with a PCR product of the *GAL-POL1* construct amplified from yeast strain NPD1 (Lemoine 2005) resulted in strain AMC308.

Haploid AMC306 is isogenic with strain MS71 (Lemoine 2005) and was created in the following steps. Starting with strain AMC296 (described above in the construction of haploid Y317), the wild-type allele of *URA3* was inserted on chromosome III at base 313553, creating strain AMC304. Then, transformation with a PCR product of the *GAL-POL1* construct amplified from yeast strain NPD1 (Lemoine 2005) resulted in strain AMC306.

Haploid Y368 is isogenic with strain MS71 (Lemoine *et al.* 2005) and was created by transformation of strain Y328 with a PCR product of the *NAT* drug resistance gene (Goldstein and McCusker 1999) targeted for insertion on chromosome III at base 169125, in between the two Ty1 elements of fragile site FS2.

Haploid AMC330 is isogenic with strain MS71 (Lemoine *et al.* 2005) and was created by transformation of strain Y317 with a PCR product of the *NAT* drug resistance gene (Goldstein and McCusker 1999) targeted for insertion on chromosome III at base 168239, such that it replaces both of the two Ty1 elements of fragile site FS2.

Haploid AMC328 is isogenic with strain YJM789 (Wei *et al.* 2007) and was created transformation of strain AMC273 with a PCR product of the *NAT* drug resistance gene (Goldstein and McCusker 1999) targeted for insertion on chromosome III at base 168239, such that it replaces the single Ty1 element that had previously been inserted.

**REFERENCES**

Goldstein, A. L., and J. H. McCusker, 1999 Three new dominant drug resistance cassettes for gene disruption in Saccharomyces cerevisiae. Yeast **15:** 1541-1553.

Lee, P. S., P. W. Greenwell, M. Dominska, M. Gawel, M. Hamilton *et al.*, 2009 A fine-structure map of spontaneous mitotic crossovers in the yeast Saccharomyces cerevisiae. PLoS Genet **5:** e1000410.

Lemoine, F. J., N. P. Degtyareva, K. Lobachev and T. D. Petes, 2005 Chromosomal translocations in yeast induced by low levels of DNA polymerase a model for chromosome fragile sites. Cell **120:** 587-598.

Storici, F., L. K. Lewis and M. A. Resnick, 2001 In vivo site-directed mutagenesis using oligonucleotides. Nat Biotechnol **19:** 773-776.

Wei, W., J. H. McCusker, R. W. Hyman, T. Jones, Y. Ning *et al.*, 2007 Genome sequencing and comparative analysis of Saccharomyces cerevisiae strain YJM789. Proc Natl Acad Sci U S A **104:** 12825-12830.
